# Redefining Dendritic Cell Vaccines: Synergistically Co‐priming DC and B Cells With Nanoparticles Loading Whole Cell Antigens Maximizes the Efficacy of DC Vaccines

**DOI:** 10.1002/advs.202510615

**Published:** 2026-02-11

**Authors:** Xiangxiang Xu, Xianlan Chen, Jin Wang, Yuhan Liu, Sidra Mustafa, Lu Diao, Rongrong Zhao, Haiyang Chi, Kang Hu, Jiashan Zhu, Jun Zhao, Mi Liu

**Affiliations:** ^1^ Department of Pharmaceutics College of Pharmaceutical Sciences Soochow University Suzhou P. R. China; ^2^ Institute of Minimally Invasive Thoracic Cancer Therapy and Translational Research Soochow University Suzhou Jiangsu P. R. China; ^3^ Research and Development Department Suzhou Ersheng Biopharmaceutical Co., Ltd Suzhou Jiangsu P. R. China; ^4^ Research and Development Department Wuxi Boston Biopharmaceutical Co., Ltd Wuxi P. R. China; ^5^ Department of Key Laboratory Affiliated Changshu Hospital of Nantong University Changshu China; ^6^ Institute of Thoracic Surgery The First Affiliated Hospital of Soochow University Soochow University Suzhou Jiangsu P. R. China; ^7^ Department of Thoracic Surgery The First Affiliated Hospital of Soochow University Soochow University Suzhou Jiangsu P. R. China; ^8^ Jiangsu Province Engineering Research Center of Precision Diagnostics and Therapeutics Development Soochow University Suzhou P. R. China

**Keywords:** cancer vaccine, B cell, dendritic cell, DC‐BC vaccine, cancer immunotherapy

## Abstract

Dendritic cells (DC) play core roles in inducing antigen‐specific T cells. However, limited effectiveness hinders their applications as vaccines. To improve the efficacy of traditional DC vaccines, this study optimized three aspects: tumor antigens, cell sources and co‐incubating molecules, and thus proposed a new DC‐BC vaccine: co‐priming B cells and DC at a precise ratio. The therapeutic efficacy was significantly improved by co‐incubating DC and B cells. Regarding tumor antigens, utilizing nanoparticles loading whole‐tumor lysates performed better than utilizing nanoparticles loading multiple neo‐antigens, or nanoparticles loading only water‐soluble lysates, or free whole‐tumor lysates. Moreover, adding IL‐15 and αPD‐L1 antibody further bettered DC‐BC vaccines. The optimal DC‐BC vaccines showed excellent therapeutic efficacy with a 100% response rate and could cure most tumor‐bearing mice on several different cancer models, including melanoma, lung cancer and orthotopic pancreatic cancer. The mechanism investigation demonstrated that several molecules, including Ticam1 (TRIF), Traf3, Mavs, and Ifnar2, were involved in promoting APC maturation and improving therapeutic efficacy of vaccines by activating innate immune pathways (TLR/NLR/RLR). In summary, this study provides a new DC‐BC vaccine that has much better therapeutic efficacy and explores the underlying mechanism of why co‐incubating DC and B cells improved the therapeutic efficacy.

## Graphical abstract

Dendritic cells (DC) vaccine is an important type of vaccines. However, the limited efficacy of DC vaccines hinders their widely application. This study redefines DC vaccines and provides a new DC‐BC vaccine that have much better therapeutic efficacy. The underlying mechanism of why co‐incubating DC and B cells improved the therapeutic efficacy is explored.

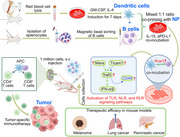

## 1 Introduction

In recent years, immunotherapy, which harnesses and enhances the body's own immune system to precisely recognize and target tumor cells, has exerted a profound influence on the paradigms of cancer treatment [1, 2, 3]. This field encompasses a wide array of modalities, including cellular therapy [4, 5], immune enhancement therapy [6], immune modulation therapy [7], monoclonal antibody therapy [8], and cancer vaccine therapy etc [9]. Among them, cancer vaccines and cellular therapy have emerged as important methods of cancer immunotherapies, owing to their remarkable advantages such as precision, personalization, long—lasting efficacy, and relatively low side—effect profiles etc [10]. Among them, dendritic cells (DC) cancer vaccine is a crucial strategy of cancer vaccines [11, 12].

DC are the most important antigen‐presenting cells (APC) to specifically activate antigen‐specific T cells, and thus DC can be developed as cellular cancer vaccines. As a highly promising type of cancer vaccines, DC vaccines exhibited some advantages [13, 14, 15]. These encompass personalized customization, superior safety profiles, enduring immune memory, outstanding synergistic effects when integrated with other treatment modalities, and broad applicability across a diverse range of cases etc [16, 17]. Currently, DC cancer vaccines are categorized into two types: DC peptide vaccines and DC gene vaccines. DC peptide vaccines include DC vaccines stimulated by tumor antigens, DC vaccines pulsed with tumor cell lysates, DC vaccines created by fusing tumor cells with DC and DC vaccines loaded with exosomes [18, 19, 20]. DC gene vaccines involve modifying DC vaccines with tumor DNA, tumor RNA and cytokines etc [19, 20]. Utilizing peripheral blood is the predominant method for the preparation of DC vaccines, primarily due to its relative ease of accessibility [21, 22].The preparation procedure typically entails the following sequential steps: (1) collecting patient's peripheral blood; (2) mononuclear cells are isolated [23, 24]; (3) subsequently, DC cells are obtained through in vitro culture and activation processes; (4) tumor antigens are co‐cultured with the activated DC cells [19]. This co‐culturing step facilitates the effective presentation of the tumor antigens to T cells by the DC cells.

The effective acquisition of tumor antigens is a critical step in DC vaccine preparation. A representative example is Sipuleucel‐T [25], the only DC vaccine currently approved by the U.S. Food and Drug Administration (FDA). This autologous product was developed by co‐culturing a patient's antigen‐presenting cells (APC) with prostatic acid phosphatase (PAP), a prostate cancer‐associated antigen, enabling the induction of T cell‐mediated immune responses to target PAP‐expressing tumor cells [26]. However, due to tumor heterogeneity [27], the identification of common tumor‐specific antigens across diverse malignancies remains challenging [28, 29], which limits the efficacy of DC cancer vaccines.

The therapeutic efficacy of traditional DC vaccines can be further improved in three aspects: (1) tumor antigens and their formulation utilized to stimulate DC vaccines; (2) the cell sources of vaccines; (3) the co‐stimulatory molecules and process of DC vaccines during co‐incubating.

The utilization of tumor lysates as antigens to prime DC represents a promising approach to prepare DC vaccines [30], which possessvarious advantages [30, 31, 32]. However, previous studies can only use the supernatant of tumor lysates as tumor antigens to stimulate DC due to the technology limitations of being unable to apply real whole tumor lysates (including both water‐soluble components and water‐insoluble components) [18, 33, 34, 35, 36]. For instance, DCVax‐L—an autologous tumor lysate‐loaded DC vaccine developed by Northwest Biotherapeutics—has demonstrated promising clinical outcomes in phase II/III glioblastoma trials [37, 38]. Nevertheless, the efficacy needs to be further improved to achieve maximized therapeutic effect by including water‐insoluble components of tumor lysates into tumor antigens [20]. Our previous studies presented a new method to solubilize water‐insoluble components and proved that using 8 M urea as a solubilizer didn't affect the immunogenicity of tumor antigens. Besides, free tumor lysates cannot penetrate cell membrane of APC and thus encapsulating tumor lysates into nanoparticles can facilitate the uptake of tumor antigens for better activation. Therefore, utilizing solubilized real whole tumor antigens, especially being encapsulated into nanoparticles (NP), to stimulate DC vaccines potentially can improve the efficacy of DC vaccines.

DC is the most important APC and DC alone can activate antigen‐specific T cells. Therefore, scientists applied DC alone as cancer vaccines. However, the vaccines based on DC alone is suffering from the problem of relatively low therapeutic efficacy. B cells are also important APC. In addition, in draining lymph nodes, B cells are involved in the process of initially activating naïve T cells to become antigen‐specific T cells. Therefore, including B cells into traditional DC cancer vaccines potentially can also improve the efficacy of cancer vaccines.

**FIGURE 1:**
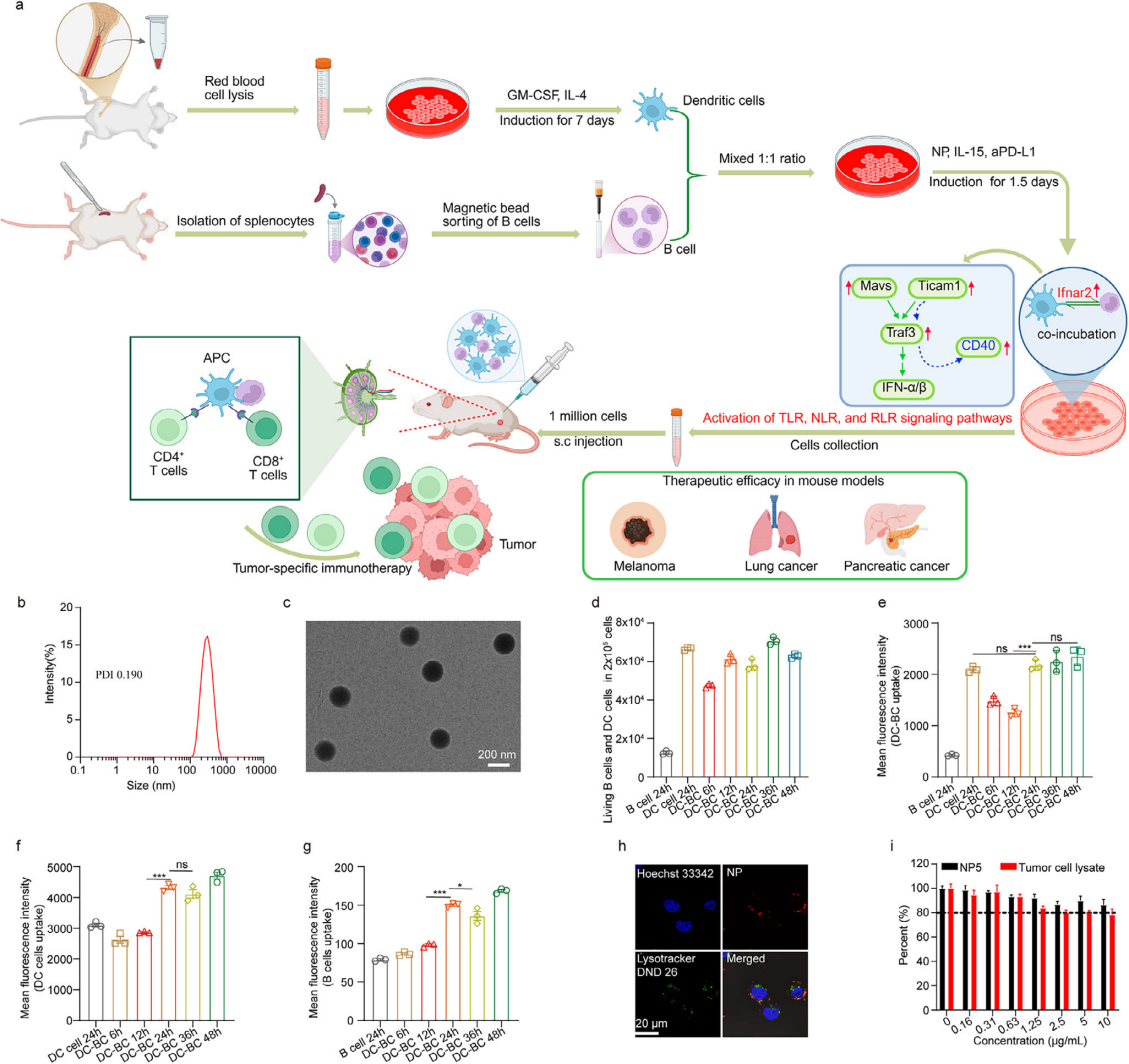
The schematic illustration of this study and the characterization of DC‐BC Vaccines. (a) The schematic illustration of improving the therapeutic efficacy of DC‐BC vaccine by co‐incubating DC and B cells, and intrinsic mechanism. (b) Hydrodynamic size (∼290 nm, PDI 0.190) of nanoparticles (NP). (c) The morphology of NP analyzed with transmission electron microscope (TEM), Scale bar, 200 nm. (d–g) The cellular uptake of rhodamine‐labeled NP by DC+B Cells (1:1), DC or B cells at different time points after co‐incubating DC and B cells with NP. (h) Subcellular distribution of NP in DC2.4 cells stained with LysoTracker DND‐26 and Hoechst 33342. Scale bar, 20 µm. (i) The investigation of potential cytotoxicity of NP and cell lysates. Data are shown as mean ± SEM (n = 3). Statistical significance (* *p* < 0.05, ** *p* < 0.01, and *** *p* < 0.001) was calculated via one‐way ANOVA with a Tukey post hoc test.

Leveraging the intrinsic capacity of DC and B cells to prime naïve T cell responses [39], along with their synergistic cross‐talk as APC, this study proposes a novel type of cellular cancer vaccine: preparing cellular cancer vaccine by co‐culturing B cells and DC at optimized ratios, which is called as DC‐BC vaccine. During in vitro co‐incubation with tumor antigens, B cells and DC mutually activate each other, and the presence of autologous tumor lysate‐loaded nanoparticles further promotes DC maturation. Additionally, some studies have demonstrated that the use of specific cytokines during DC preparation can significantly enhance the efficacy of DC vaccines [40, 41]. Therefore, this study also explored various cytokines in combination with nanoparticles to co‐prime DC and B cells, aiming to further improve the efficacy of DC‐BC vaccines. In vivo studies on tumor‐bearing mice demonstrated that this approach exhibits superior therapeutic efficacy, and the intrinsic mechanism of improved efficacy was illustrated. In summary, herein provides a new DC‐BC vaccines based on both DC and B cells primed with nanoparticles loading whole‐tumor antigens (Figure 1a).

### 1.1 Preparation and In Vitro Characterization of Nanoparticles and Cellular Vaccines

Most free proteins cannot penetrate cell membranes and cannot be uptake by APC, and thus free antigens or free tumor lysates cannot efficiently activate DC. Therefore, nanoparticles were applied to encapsulate tumor antigens for better uptake and activation. Considering tumor cells and tumor antigens are highly heterogeneous, utilizing whole tumor lysates (including both water‐soluble components and water‐insoluble components) possess various advantages in inducing more diverse antigen‐specific T cells and having better therapeutic efficacy.

Nanoparticles loaded with whole‐tumor antigens are based on our previous studies. Herein, an optimized nanoparticle (NP5), was applied illustrate the preparation and characterization of NP. In addition, this NP5 was subsequently incubated with DC/B cells to prepare DC vaccines or DC‐BC vaccines. Dynamic light scattering (DLS) analysis revealed a hydrodynamic particle size primarily centered around 290 nm (Table S1, Figure 1b), accompanied by a polydispersity index (PDI) of 0.190. Transmission electron microscope (TEM) study confirmed their spherical morphology, with a size of 200 ± 10 nm (Figure 1c). And then, the uptake of these nanoparticles by APC was examined, utilizing 0.5 mg/mL of rhodamine‐labeled NP in uptake experiments with a 1:1 mixture of bone marrow‐derived dendritic cells (BMDCs) and B cells. The results demonstrated that the uptake of NP by both B cells and BMDCs was time‐dependent, plateauing after 24 h (Notably, BMDCs exhibited a 27.4‐fold higher uptake compared to B cells, Figure 1d–g). Confocal subcellular localization analysis revealed that the NP were mainly confined within endosome‐lysosomal subcellular compartments. Some others managed to escape from the endosome‐lysosome system and entered into the cytoplasm (Figure 1h). In the endosome‐lysosomes or cytoplasm, antigens can be processed and presented to MHC II and MHC I molecules. Cytotoxicity experiments revealed NP loaded with whole‐tumor lysates have no cytotoxicity at concentrations below 10 mg/mL. However, when free tumor tissue lysates (containing an equivalent amount of nanoparticle‐loaded lysates) was administered at concentrations exceeding 2.5 µg/mL, approximately 20% cytotoxicity was observed (Figure 1i). The toxicity associated with the free tumor tissue lysates could be attributed to components that can bind with receptors on the surface of cell membranes.

### 1.2 DC Can be Stimulated With NP Loading Whole‐Tumor Antigens in Time‐Dependent and Concentration‐Dependent Manner

To elucidate the impact of concentrations of the NP (NP5) loaded with whole tumor antigens, the efficacy of stimulating bone marrow‐derived dendritic cells (BMDCs) with NP5 was conducted in the condition of co‐incubating BMDCs with NP for 24 h. The experimental outcomes revealed that, within a NP concentration range of 0.3 to 0.8 mg/mL, the survival rate of bone marrow‐derived monocytes augmented by roughly 30% in comparison to the PBS control group (Figures S1–S3a). Furthermore, BMDC survival rates were notably higher when the NP concentration was maintained between 0.1 and 0.5 mg/mL (Figure S3b,c). Intriguingly, when the NP concentration surpassed 0.3 mg/mL, the number of mature BMDCs was 2.1 times greater than that of the PBS group (Figure S3d). When the concentration of NP is in the range of 0.3–2 mg/mL, the expression of MHC‐II in BMDCs was at least 15% higher compared to the PBS group (Figure S3e). However, within a NP concentration range of 0.3 to 1.0 mg/mL, MHC‐I expression exhibited a 59% increase compared to the PBS group (Figure S3f). Considering the favorable performance of all immune indicators at a concentration of 0.5 mg/mL, this concentration was selected for further priming of BMDCs and the measurement of BMDC‐related immune indicators at different time points. The experimental results demonstrated that the survival rate of BMDCs incubated with NP for 48 h was comparable to that of the PBS group, with the highest survival rate observed after 24 h of incubation, representing an approximately 8% increase compared to the PBS group (Figure S3g). Among the 500 000 cells collected, the survival rate of BMDCs increased over time, possibly due to the gradual death of monocytes, thereby enhancing the purity of BMDCs (Figure S3h). The number of CD11c^+^ cells and mature BMDCs increased from 1 to 24 h and then declined from 24 to 72 h (Figure S3i,j). The expression of MHC‐II was doubled compared to the PBS group during the 4 to 36 h of NP co‐incubation (Figure S3k). There was no significant change in MHC‐I expression after 4 h of NP co‐incubation (Figure S3l). Based on these results, it can be concluded that incubating BMDCs with NP loaded with whole‐tumor antigens at a concentration of 0.5 mg/mL for 24 h is an optimal condition.

### 1.3 Whole Tumor Antigens Are Needed and Crucial for Preparing DC‐BC Vaccines

**FIGURE 2:**
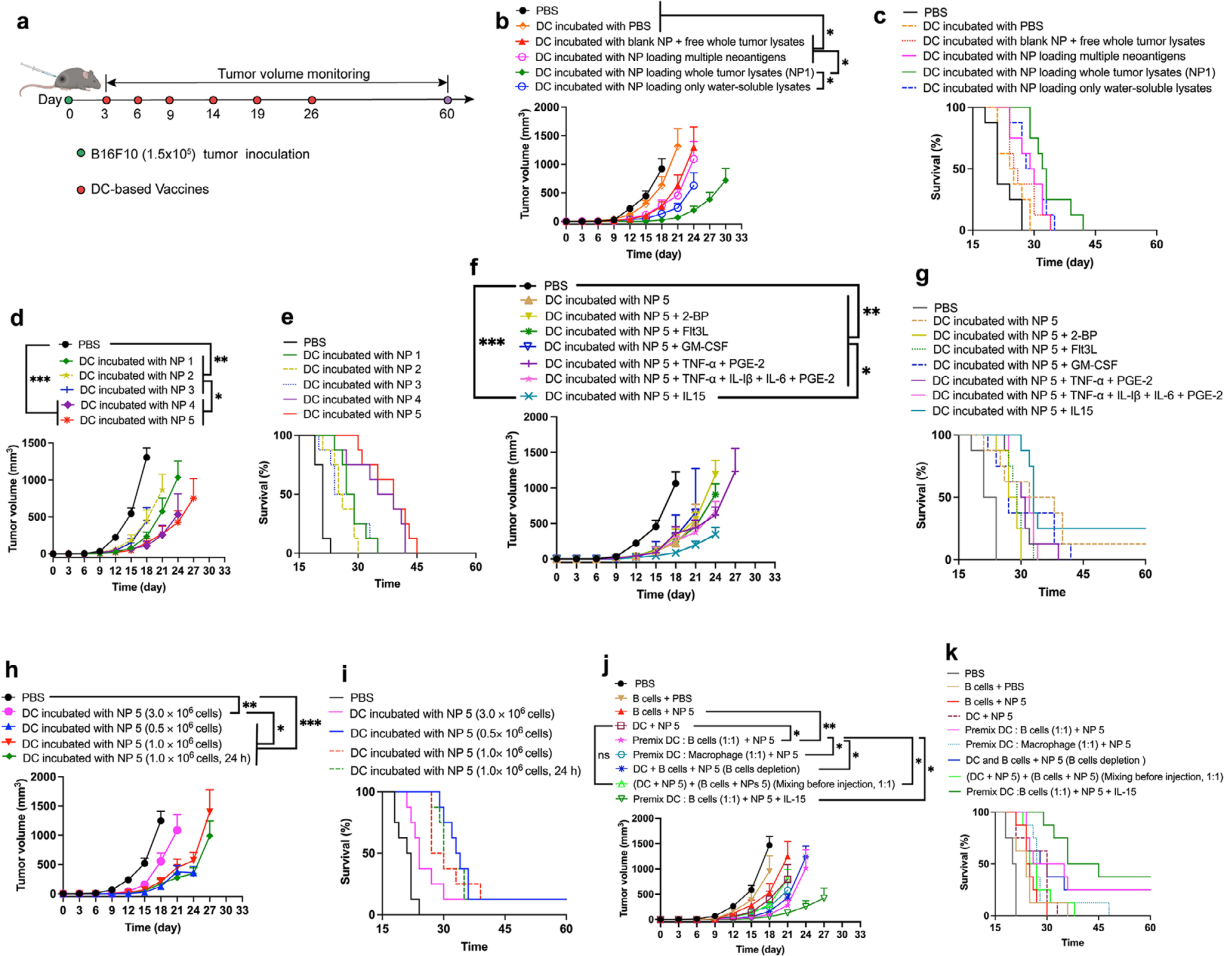
The therapeutic efficacy of DC or DC‐BC vaccine on tumor‐bearing B16F10 mouse model. (a) The scheme of tumor inoculation and vaccine treatment (This treatment regimen is applicable to (b,‐k). (b,c), The tumor growth curve and survival curve of tumor‐bearing B16F10 mice treated with DC vaccines stimulated with different forms of whole tumor cell lysates. (d,e), The tumor growth curve and survival curve of tumor‐bearing B16F10 mice treated with DC vaccines stimulated with different NP. (f,g), The tumor growth curve and survival curve of tumor‐bearing B16F10 mice treated with DC vaccines stimulated with NP + different cytokines. (h,i), The tumor growth curve and survival curve of tumor‐bearing B16F10 mice treated with different cell numbers of DC vaccines. (j,k), The tumor growth curve and survival curve of tumor‐bearing B16F10 mice treated with different DC or DC‐BC vaccines. Data are shown as mean ± SEM (n = 8). Statistical significance (* *p* < 0.05, ** *p* < 0.01, and *** *p* < 0.001) was calculated via one‐way ANOVA with a Tukey post hoc test.

Subsequently, DC induced under the above conditions were used in therapeutic studies on tumor‐bearing B16F10 mice, and the treatment scheme is shown in Figure 2a. Groups treated with pure DC, DC stimulated with blank NP + free whole tumor lysates, DC stimulated with NP loaded with multiple neo‐antigens, and DC stimulated with NP loaded with only water‐soluble lysates had mild inhibitory effects on the tumor growth. Meanwhile, the group treated with DC vaccines stimulated with NP showed a tumor inhibition rate of 97.2%, with a survival time increased by 10 days compared to the PBS group (Figure 2b,c).

Tumor tissue lysates contain different types tumor‐specific antigens (TSA, neo‐antigens) and tumor associated antigens (TAA). Therefore, whole tumor tissues lysate is the idea source of tumor antigens. However, due to the limitations of technologies, only water‐soluble components in tumor tissues lysates (the supernatant after centrifuge of tumor tissue lysates) can be used to prepare DC vaccines in previous studies [18, 33, 34, 35, 36]. We developed a method (utilizing 8 M urea) that can solubilize both water‐soluble components and water‐insoluble components in tumor tissue lysates, and thus whole tumor tissue lysates can be encapsulated into nanoparticles and be utilized to stimulate DC, for subsequently activating tumor antigen‐specific T cells [32, 42, 43, 44, 45]. By this method, for the first time, real whole‐tumor antigens can be utilized to prepare cancer vaccines.

These results demonstrated that DC vaccines stimulated with NP loading whole tumor antigens (both water‐soluble antigens and water‐insoluble antigens) exhibited superior therapeutic efficacy, comparing with DC vaccines stimulated with NP loading only water‐soluble antigens and DC vaccines stimulated with NP loading multiple neo‐antigens. This is due to more heterogeneous tumor antigens can induce DC vaccines that carrying more diverse tumor antigens and thus stimulated more diverse antigen‐specific T cells. More diverse antigen‐specific T cells can recognize and kill tumor cells more efficiently. These studies illustrated that encapsulating tumor antigens into nanoparticles and including real whole‐tumor antigens (both water‐soluble components and solubilized water‐insoluble components) is crucial to maximize the therapeutic efficacy of cellular vaccines (DC vaccines or DC‐BC vaccines).

### 1.4 Treatment of Melanoma‐Bearing Mice With DC Vaccines or DC‐BC Vaccines Primed With NP Loading Whole Tumor Antigens

Different DC vaccines, stimulated with NP prepared with different processes and adjuvants, were compared in terms of therapeutic efficacy. All these 5 different NP loading whole‐tumor tissue lysates as tumor antigens. NP1 loaded poly (I:C) as adjuvants and whole tumor lysates solubilized with 8 M urea. NP2 loaded CpG7909 + CpG 2395 as adjuvants and whole tumor lysates solubilized with 8 M urea. NP3 loaded lysates of BCG as adjuvants and whole tumor lysates solubilized with 8 M urea. NP4 loaded poly (I:C) + CpG 7909 + CpG2395 as adjuvants and whole tumor lysates solubilized with 6 M guanidine hydrochloride. NP5 loaded poly (I:C) + CpG7909 + CpG2395 as adjuvants and whole tumor lysates solubilized with 8 M urea. Notably, all DC primed with different NP demonstrated certain inhibitory effects on tumor growth (Figure 2d,e). NP (NP 4 and NP 5) loaded with poly (I:C) and CpG showed the best efficacy. In addition, NP5 loading whole tumor lysates solubilized with 8 M urea showed similar therapeutic efficacy with NP4 loading with whole tumor lysates solubilized with 6 M guanidine hydrochloride.

The immune microenvironment within the body can significantly impact the activation of DC vaccines and subsequent therapeutic efficacy of DC vaccines. Cytokines or other chemicals are the major force that can influence the immune microenvironment. Therefore, the influences of different cytokines or chemical in affecting DC activation were compared. These cytokines and chemicals include GM‐CSF, TNF‐α/PEG‐2, FLT3‐L, IL‐15, and TNF‐α/PEG‐2/IL‐6/IL‐1. The experimental results revealed that the tumor inhibition effect of the GM‐CSF group was diminished in comparison to the NP‐alone group. Both the TNF‐α/PEG‐2 group and the TNF‐α/PEG‐2/IL‐6/IL‐1 group exhibited tumor inhibition effects that were comparable to those observed in the BMDC group induced by NP. However, the IL‐15 group demonstrated a significant improvement in therapeutic efficacy [46]. This indicates that IL‐15 can effectively promote DC maturation and thus enhance the capacity of DC vaccines to activate antigen‐specific T cells (Figure 2f,g).

The impact of vaccines priming time and the utilized number of DC cells on therapeutic efficacy were investigated. Experimental results revealed that the worst therapeutic outcome occurred when 3 million DC were administered in a single dose. When the number of administered DC was reduced to 0.5 million, the tumor inhibition rate reached 89.8%. With 1 million cells administered, the tumor inhibition rate was 86.6%, indicating that the therapeutic effects were comparable between these two cell quantities. When the DC stimulation time was extended from 24 to 36 h, the tumor inhibition rate was 83.6%, representing a slight reduction compared to the 24‐h induction period (Figure 2h,i). Though exhibited therapeutic effect, the therapeutic efficacy of DC can be further improved.

DC, B cells and macrophages can serve as antigen‐presenting cells within the body. In addition, in draining lymph nodes, DC and B cells have complex interactions during the activation of DC with tumor antigens. Therefore, we proposed that the co‐incubation of two distinct antigen‐presenting cell types might effectively improve therapeutic efficacy of cancer vaccines. Herein, we embarked on an exploration of the tumor‐inhibitory effects achieved by mixing DC in equal proportions with either B cells or macrophages. The experimental findings revealed a tumor inhibition rate of 35.2% when employing B cells alone, 79.8% when DC were combined with macrophages in equal measures, and a notable 93.2% when DC cells were mixed in equal proportions with B cells. These data demonstrated that the interactions between DC and B cells are needed during the process of antigen presentations in DC.

The therapeutic efficacy was further improved when DC cells + B cells were further co‐incubated with IL‐15, and the tumor inhibition rate ascended to 96.2%. Conversely, when DC were blended in equal proportions with B cells while concurrently depleting B cells in vivo, the tumor inhibition rate plummeted to 60.0%, indicating the pivotal role played by B cells in the efficacy of DC vaccines.

Furthermore, When DC and B cells were individually incubated with NP and subsequently mixed in equal proportions prior to injection, the therapeutic effect diminished, yielding a tumor inhibition rate of 79.8% (Figure 2j,k). In addition, applying the mixture of DC and B cells showed better therapeutic efficacy than utilizing the mixture of DC and macrophages, indicating the necessary of including B cells into the vaccine formulation. Hence, it is imperative that both DC and B cells are needed when undergoing co‐incubation with NP. These data verify that, to obtain optimal DC‐ BC cancer vaccines, co‐incubating DC and B cells with tumor antigens, which is considerably different with the current DC vaccine's preparation method.

### 1.5 Proteomics Study on the Mechanism of Enhanced Therapeutic Efficacy by Co‐Culturing B Cells and DC

**FIGURE 3:**
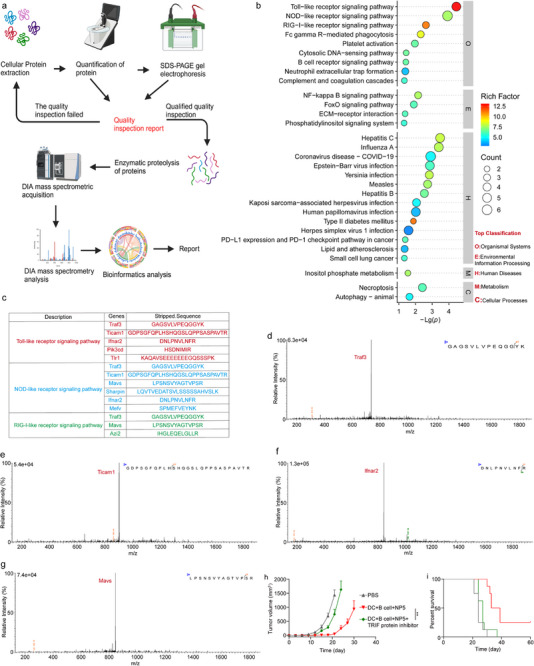
Exploring the mechanism of enhanced therapeutic efficacy resulting from co‐incubation of B cells and DC through proteomic analysis. (a) Sample preparation and detection workflow in proteomic analysis. (b) The results of KEGG analysis. (c) Significantly altered signaling pathways identified by KEGG analysis, along with enriched proteins and their corresponding peptide sequences. (d–g) Secondary peptide mapping of shared proteins in three signaling pathways. (h,i) Tumor volume and survival curve of tumor‐bearing B16F10 mouse model treated with DC‐BC vaccine incubated with TLR‐TRIF protein inhibitor (The treatment regimen is the same as that in Figure 2a). Data are shown as mean ± SEM (n = 8). Statistical significance (** *p* < 0.01) was calculated via one‐way ANOVA with a Tukey post hoc test.

To explore the mechanisms underlying the interactions between DC and B cells during co‐incubation with NP loading whole tumor antigens, a proteomic analysis was conducted after co‐incubating these two cell types with NP loading whole tumor antigens. The experimental findings unveiled that co‐incubation effectively stimulated innate immune pathways in cells, notably the Toll‐like receptor signaling pathway, NOD‐like receptor signaling pathway, and RIG‐I‐like receptor signaling pathway (Figure 3a,b). These pathways were enriched with 5, 6, and 3 proteins, respectively, emerging as the most prominent among all the pathways analyzed (Figure 3c).

MAVS is a key protein in the RIG‐I and NOD‐like signaling pathways, while Ticam1 (TRIF) plays a crucial role in the Toll‐like and NOD‐like signaling pathways. Traf3 is a shared protein in the Toll‐like, RIG‐I, and NOD‐like signaling pathways [47]. The significant increase in MAVS, Ticam1 (TRIF), and the downstream shared protein Ticam1 (TRIF) effectively activates the innate immune pathway, promoting the secretion of type I interferons [48]. Meanwhile, co‐culturing DC cells with B cells significantly enhances the expression of the Ifnar2 protein. As an interferon receptor, the increased expression of Ifnar2 improves cellular sensitivity to interferons [49], thereby promoting the maturation of DC cells and B cells. Additionally, the activation of the Toll‐like signaling pathway by the increased levels of Ticam1 (TRIF) and Traf3 effectively upregulates the expression of CD40 on the surface of DC cells and B cells [50]. The increase in CD40 effectively activates T cells, further enhancing the immune response.

Furthermore, we identified the corresponding peptide sequences and their secondary spectra for further validation (Figure 3d–g). The activation of these innate immune pathways efficiently promotes the maturation and activation of immune cells, exhibiting superior effects in comparison to adjuvants. This suggests that the combination of these two cell types during the APC activation can mutually activate each other, thereby significantly enhancing their maturation and vaccination capabilities. Highly mature antigen‐presenting cells (APC) can dramatically improve antigen‐specific T cell activation, ultimately leading to a substantial enhancement in therapeutic outcomes.

The proteomics research highlighted that the amalgamation of B cells and DC cells potently activates innate immune pathways. To delve deeper into this phenomenon, we employed a TLR‐TRIF protein inhibitor to impede the innate immune pathways in both cell types. The experimental results indicated that the tumor inhibition rate of the DC‐BC vaccine prepared with the addition of the TRIF protein inhibitor was 40.9%, whereas the inhibition rate for the group without the inhibitor was a substantial 95.5%. These studies demonstrated why the significant enhancement in therapeutic efficacy achieved by the mixture of the two cell types (Figure 3h,i).

### 1.6 Exploration of Immune Indicators in BMDCs and B Cells Co‐Primed With NP at Different Concentrations and Different Durations

To explore the impact of NP incubation concentration on the therapeutic efficacy of DC‐BC vaccines, BMDCs and B cells (mixed in a 1:1 ratio) were co‐incubated with NP at different concentrations for a duration of 36 h. Subsequently, the cells were collected and stained for flow cytometry analysis. The studies revealed that the overall cell survival rate in the NP‐treated groups surpassed that of the PBS control group, with a notable 1.4‐fold enhancement observed at a concentration of 0.5 mg/mL (Figures S4 and S5a). Within the mixed cellular milieu, the count of BMDCs augmented progressively with the escalation of NP concentration (Figure S5b). Conversely, the count of CD11C^+^ cells exhibited an upward trend within the concentration bracket of 0.1–0.5 mg/mL but dipped below the PBS group's level when the concentration surpassed 0.8 mg/mL (Figure S5c). Across all concentrations, the number of mature DC cells was elevated compared to the PBS group, with the most favorable concentration being 0.5 mg/mL, demonstrating a 2.6‐fold increase relative to the PBS group (Figure S5d). The mean fluorescence intensity of MHC‐I on the DC cells surface at various concentrations ranged between 4 and 5 times that of the PBS group (Figure S5e). Within a NP concentration range of 0.3 to 1.0 mg/mL, the percentage expression of MHC‐II on the DC cell surface augmented by more than 37% (Figure S5f). The expression of CD40L on the DC cell surface incremented with the NP concentrations within the range of 0.1–2.0 mg/mL, peaking at 4.3 times the expression level of the PBS group (Figure S5g). The expression of CD40 on the DC cell surface escalated with concentration within the range of 0.1–0.8 mg/mL but declined within the range of 1.0–2.0 mg/mL. However, it remained elevated at 13.2 times the level of the PBS group, with a peak expression of 16.7 times that of the PBS group (Figure S5h).

In the mixed cellular population, the co‐incubation of B cells with NP facilitated an enhancement in B cell survival, with a survival rate increase of over 38% within the concentration range of 0.8–2.0 mg/mL (Figure S5i). The number of B220^+^ cells increased with the concentration within the range of 0.1–0.8 mg/mL, but declined slightly when the concentration exceeded 0.8 mg/mL, achieving a survival rate 1.4 times that of the PBS group at 0.8 mg/mL (Figure S5j). The percentage of CD80/CD86‐positive B cells increased with the NP concentration within the range of 0.1–1.5 mg/mL, with a slight decrease at 2.0 mg/mL. The expression level was more than double that of the PBS group within the concentration range of 0.3–1.5 mg/mL (Figure S5k,l). The mean fluorescence intensity of MHC‐I on the surface of B cells at various concentrations did not correlate with the concentration (Figure S5m). The percentage expression of MHC‐II on the surface of B cells increased by over 18% when the NP concentration ranged from 0.1 to 2.0 mg/mL (Figure S5n). The expression of CD40 on the surface of B cells increased with the NP concentration within the range of 0.1–0.8 mg/mL, while the expression on the surface of DC cells declined within the range of 1.0–2.0 mg/mL. At 0.8 mg/ml, the expression level was 4.6 times that of the PBS group (Figure S5o). The expression of CD40L on the surface of B cells increased with the NP concentration within the range of 0–0.3 mg/mL but declined within the range of 0.3–2.0 mg/mL, with the highest expression level being 1.3 times that of the PBS group (Figure S5p). Considering the results of various indicators for BMDCs and B cells with different NP concentrations, a concentration of 0.5 mg/mL of NP was selected for a 36 h NP co‐incubation time.

**FIGURE 4:**
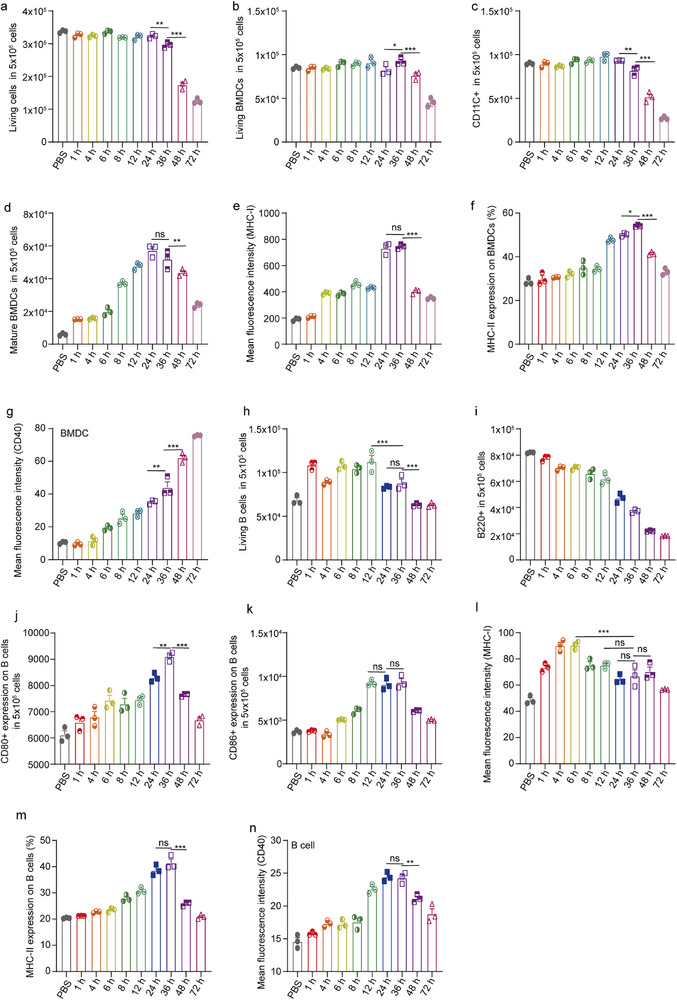
The expressions of different surface markers when BMDC are co‐incubated with B cells (1:1 premixed) and 0.5 mg/mL NP5 nanoparticles at different time points (in 500 000 cells). (a) The quantifying analysis of all survival cells. (b) The quantifying analysis of survival BMDCs. (c) The quantifying analysis of CD11c^+^ BMDCs. (d) The quantifying analysis of CD80^+^ and CD86^+^ BMDCs. (e) The analysis of MHC‐I relative fluorescence intensity statistics of BMDCs. (f) The analysis of MHC‐II^+^ BMDCs. (g) The analysis of CD40^+^ relative fluorescence intensity statistics of BMDCs. BMDCs and B cells were co‐incubated with 0.5 mg/mL NP5 nanoparticles (1:1 premixed) at different time points (500 000 cells). (h,i) The analysis of survival B cells after co‐incubation. (j,k) The analysis of CD80^+^ B cells and CD86^+^ B cells. (l) The analysis of MHC‐I relative fluorescence intensity statistics of B cells. (m) The analysis of MHC‐II expression percentage of B cells. (n) The analysis of CD40^+^ relative fluorescence intensity statistics of B cells. Data are shown as mean ± SEM (n = 3). Statistical significance (* *p* < 0.05, ** *p* < 0.01, and *** *p* < 0.001) was calculated via one‐way ANOVA with a Tukey post hoc test.

Utilizing this selected concentration, an investigation on the impact of co‐incubation time with NP were conducted. The resulsts revealed that, within the first 36 h, there was no notable disparity in the overall cell survival rate between the NP treated groups and the PBS control group. Nevertheless, upon exceeding 36 h, the overall cell survival rate in the NP treated group plummeted to less than 51% of that observed in the PBS group (Figure 4a). In mixed cellular populations, both the count of BMDCs and CD11C^+^ cells exhibited no significant variations compared to the PBS group within the initial 36 h of incubation. However, between 48 and 72 h, these counts dipped below 57% of the levels recorded in the PBS group (Figure 4b,c). The quantity of mature DC augmented progressively during the 1 to 36 h of NP co‐incubation, culminating in a peak at 24 to 36 h, which was 9.6 times higher than that of the PBS group. Subsequently, from 36 to 72 h, the number of mature cells decreased with time (Figure 4d). The trends in the mean fluorescence intensity of MHC‐I and the expression of MHC‐II mirrored the maturity trajectory of DC, with peak values at 36 h being 3.9 times and 1.9 times those of the PBS group, respectively (Figure 4e,f). Furthermore, the expression of CD40 on the DC escalated with incubation time, ultimately reaching a level 7.5 times higher than that of the PBS group at 72 h (Figure 4g).

In mixed cellular cultures, the survival rate of B cells during the 1 to 72 h of was consistently higher than that of the PBS control group, peaking at 12 h with a count 1.65 times greater than the PBS group (Figure 4h). The number of B220^+^ cells decreased as the incubation time increased (Figure 4i). The trend in CD80/CD86 cell counts mirrored the time course, increasing with incubation time from 1 to 36 h and then declining between 36 and 72 h, yet remaining above the levels observed in the PBS group. Peak values at 36 h were 1.5 times and 2.5 times those of the PBS group for CD80 and CD86, respectively (Figure 4j,k). As the incubation time extended, the mean fluorescence intensity of MHC‐I on the surface of B cells was consistently above 1.5 times that of the PBS group, reaching a peak of 1.86 times the PBS group level at 6 h (Figure 4l). The expression of MHC‐II on the surface of B cells increased from 1 to 36 h and then decreased between 36 and 72 h, with a peak expression that was twice that of the PBS group (Figure 4m).

In addition, the expression of CD40 on the surface of B cells increased with incubation time from 1 to 36 h, reaching a 4.2‐fold increase compared to the PBS group at 36 h. At 48 and 72 h, the expression of CD40 on B cells decreased with time but still remained above the PBS group level by 1.6 times (Figure 4n). Based on the results of co‐incubation BMDCs and B cells with NP at different concentrations and for different times, 0.5 mg/mL of NP and 36 h co‐incubation performed best. Consequently, 0.5 mg/mL of NP and 36 h co‐incubation time were selected as conditions for further in vivo studies.

### 1.7 Co‐incubating B Cells With DC and Antigens Can Improve the Activation of DC and T Cells

To further illustrate the differences in therapeutic efficacy of different vaccines on tumor‐bearing mice, DC vaccines, B cell vaccines, DC‐BC vaccines and DC‐DM (DC + macrophages) vaccines were investigated in activating DC and T cells. The results showed that, after 36 h of co‐incubating with nanoparticles, the survival rate of DC, the percentages of mature DC, MHC‐I expression, and MHC‐II expression were all significantly increased in DC‐BC vaccines group (Figure S6a–e). Furthermore, the proliferation and activation percentages of naïve T cells were measured and the results indicated that DC‐BC vaccines promoted T cell proliferation and activation in vitro, compared with DC‐DM vaccines and DC vaccines. (Figure S6f–h). Furthermore, the DC‐BC vaccines showed better efficacy in activating T cells in vivo （Figure S6i,j）. In summary, comparing with DC alone vaccine or DC+ macrophages vaccine, the DC‐BC vaccine demonstrated better efficacy in the activation of DC and T cells, which is a potential reason of improved therapeutic efficacy.

### 1.8 The Treatment of Melanoma‐bearing Mice With DC‐BC Vaccines Prepared From the Mixture of DC and B Cells

**FIGURE 5:**
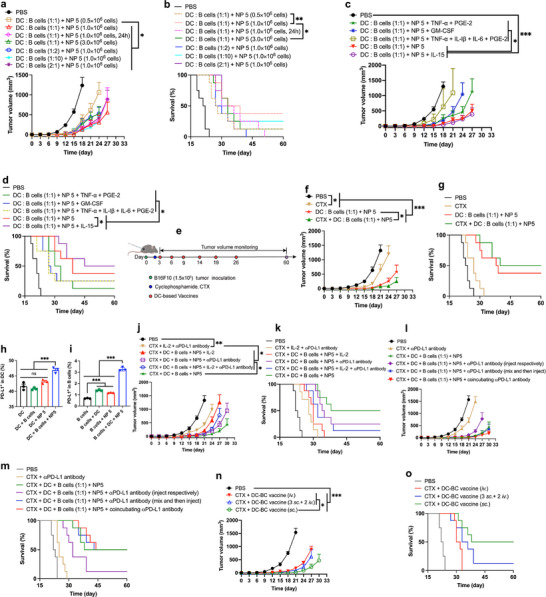
The therapeutic efficacy of DC‐BC vaccines prepared by different strategies on tumor‐bearing B16F10 mouse model. The treatment regimen in (a—d) is the same as that in Figure 2a. (a,b) The tumor growth and survival curve of tumor‐bearing B16F10 mice treated with DC‐BC vaccines at different cell numbes or different ratioes of DC and B cells; (c,d) The tumor growth and survival curve of tumor‐bearing B16F10 mice treated with DC‐BC vaccines prepared with different cytokines. (e) The dosing regimen of co‐administration cyclophosphamide with DC‐BC vaccines. (f,g) The tumor growth and survival curve of tumor‐bearing B16F10 mice treated with DC‐BC vaccines and cyclophosphamide. (h,i) Percentage of PD‐L1 expression on BMDC and B cells after co‐induction with NP (BMDC: B cells =1:1). (j,k) The tumor growth and survival curve of tumor‐bearing B16F10 mice treated with DC‐BC vaccines, cyclophosphamide and IL‐2 or aPD‐L1 antibody. (l,m) The tumor growth and survival curve of tumor‐bearing B16F10 mice treated with DC‐BC vaccines and different aPD‐L1 antibody administration methods. (n,o) The tumor growth and survival curve of tumor‐bearing B16F10 mice treated with DC‐BC vaccines at different administration routes. iv., intravenous; sc., subcutaneous. Data are shown as mean ± SEM (n = 8). Statistical significance (* *p* < 0.05, ** *p* < 0.01, and *** *p* < 0.005) was calculated via one‐way ANOVA with a Tukey post hoc test.

Based on the aforementioned exploration of the optimal incubation conditions for cellular vaccine preparation, the in vivo therapeutic effects of cellular vaccines prepared from a mixture of DC and B cells were systematically investigated by administering different numbers of cells to mice. The studies revealed that administering 500 000 DC cells per mouse resulted in a tumor inhibition rate of 70% and a survival duration of 32 days. When the dose was increased to 3 million DC cells per mouse, the tumor inhibition rate climbed to 78.9%, accompanied by a survival period of 35.5 days. However, the most potent therapeutic effect was observed when 1 million DC cells were administered per mouse, yielding a tumor inhibition rate of 89.6% and a cure rate of 37.5%. Notably, when the incubation period of cells with NP was reduced from 36 to 24 h, the cure rate plummeted to 0%. Additionally, adjustments to the mixing ratio of BMDCs to B cells (from 1:1 to 1:2, 1:10, and 2:1) led to decreases in both tumor inhibition rates and survival durations. Therefore, the optimal protocol for DC vaccine preparation was determined to be a 1:1 mixture of BMDCs and B cells incubated with NP for 36 h, followed by the administration of 1 million DC + B cells (Figure 5a,b).

Moreover, we examined the effects of incorporating various cytokines together with NP to prepare DC vaccines at a 1:1 blend of BMDCs and B cells. The findings indicated that the cocktail approach (employing four cytokines) did not exhibit a significant difference in therapeutic efficacy compared to the control group. The inclusion of TNF‐α with either PEG‐2 or GM‐CSF resulted in tumor inhibition rates that were lower than those observed in the NP‐only group (89.2%). However, when nanoparticles were paired with IL‐15 to induce DC‐BC vaccines, the tumor inhibition rate soared to 96.1%, and the cure rate reached 37.5%, marking a substantial enhancement in efficacy (Figure 5c,d).

Previous studies reported that low‐dose cyclophosphamide can effectively decrease the level of T_reg_ cells in vivo and enhance the efficacy of DC vaccines [51, 52]. Therefore, a study was conducted to explore the therapeutic efficacy of combining low‐dose cyclophosphamide (100 mg/kg) with DC‐BC vaccines in a B16F10 subcutaneous tumor model. The dosing regimen of this study is illustrated in Figure 5e. The results demonstrated that the tumor inhibition rate was improved from 89.2% to 95.8% when cyclophosphamide was used together with DC‐BC vaccines (Figure 5f,g).

Multiple studies have established that the in vitro preparation of DC vaccines results in an elevated expression of PD‐L1 on their surface, consequently diminishing their therapeutic effectiveness. Hence, an initial step was to ascertain the PD‐L1 expression levels of DC vaccines induced by nanoparticles. The experimental findings revealed that the PD‐L1 expression on the surfaces of isolated DC and B cells we 53.4% and 1.2%, respectively, which augmented to 60.8% and 4.9% upon the incubation with nanoparticles. When DC and B cells were combined, the PD‐L1 expression levels on the surfaces of DC and B cells approximated 53.3% and 2.7%, respectively, which surged to 62.3% and 22.7% following the addition of nanoparticles to the DC‐B cells mixture (Figure 5h,i, Figure S7). Therefore, to inhibit PD‐L1 expression, the DC‐BC vaccine was prepared with the addition of aPD‐L1 antibody. In addition, to stimulate the proliferation of DC‐activated antigen‐specific T cells in blood, IL‐2 (10000U) was administered every 2 days post‐DC vaccine injection for a cumulative nine doses. Nonetheless, the treatment of tumor‐bearing mice indicated that the combining DC‐BC vaccine with IL‐2 didn't show a synergistic effect, with the tumor inhibition rate actually dipping to 86.4%. The triplet combination of DC‐BC vaccine/IL‐2/aPD‐L1 antibody yielded a tumor inhibition rate of 91.5%, which surpassed that of IL‐2 alone but fell short of the DC‐BC vaccine monotherapy (Figure 5j,k). To delve into synergistic approaches for the combination of aPD‐L1 antibody and DC‐BC vaccines, the therapeutic efficacy of three combination strategies was assessed: (1) co‐incubating DC and B cells with nanoparticles and aPD‐L1 antibody during activating APC; (2) pre‐administrating aPD‐L1antibody prior to DC‐BC vaccine injecting in mice; and (3) simultaneously utilizing DC‐BC vaccine and aPD‐L1 antibody. The results demonstrated that co‐incubating DC and B cells with nanoparticles and aPD‐L1 during activating showed the highest efficacy. The remaining treatment modalities exhibited no notable divergence compared to nanoparticle monotherapy (Figure 5l,m).

Considering DC cells' potent ability to activate and expand T cells, a question arises: can DC‐BC vaccines be directly infused into the bloodstream to stimulate tumor antigen‐specific T cells and promote their proliferation? One conceivable strategy could involve an initial phase of three subcutaneous administrations of cellular vaccines to activate tumor antigen‐specific T cells, enabling them to enter the bloodstream via lymphatic circulation, followed by three intravenous injections aimed at reactivating these T cells and preventing their apoptosis due to the absence of stimulatory signals. Based on this rationale design, an antitumor experiment was designed.

The data revealed that direct intravenous administration of DC‐BC vaccines resulted in a tumor inhibition rate of 85.2%, whereas the regimen consisting of three subcutaneous injections followed by three intravenous injections yielded a tumor inhibition rate of 83.0%. Notably, both of these rates fell short of the 95.4% achieved with six subcutaneous injections. Consequently, subcutaneous administration of such cellular vaccines demonstrated superior therapeutic efficacy (Figure 5n,o). These results indicated that home back to draining lymph nodes (DLN) is crucial for DC‐BC vaccines activating tumor antigen‐specific T cells.

### 1.9 No Obvious Toxicities Were Observed on Tumor‐Bearing Mice Treated With DC‐BC Vaccines

Furthermore, we established a subcutaneous B16F10 tumor mouse model and administered DC‐BC vaccines six times according to the above regimen. Blood and major organs were collected 48 h post the final administration for blood biochemistry analysis and hematoxylin and eosin (H&E) staining. The results of liver function, blood tests for Alanine Aminotransferase (ALT), Aspartate Aminotransferase (AST), Alkaline Phosphatase (ALP), Total Bilirubin (TBIL), and Lactate Dehydrogenase (LDH), revealed no obvious toxicities. In addition, total Protein (TP) and Albumin (ALB) values showed similar results. Renal function was evaluated through blood tests for Creatinine (CREA), Blood Urea Nitrogen (BUN), and Uric Acid (UA). The studies demonstrated that following vaccine treatment, all indices of liver and renal function remained within normal ranges (Figure S8). H&E staining showed no evidence of damages to vital organs in mice post‐treatment, underscoring the exceptional safety profile of DC‐BC vaccines (Figure S9).

### 1.10 The Therapeutic Efficacy of DC‐BC Vaccines Stored at Frozen Form

**FIGURE 6:**
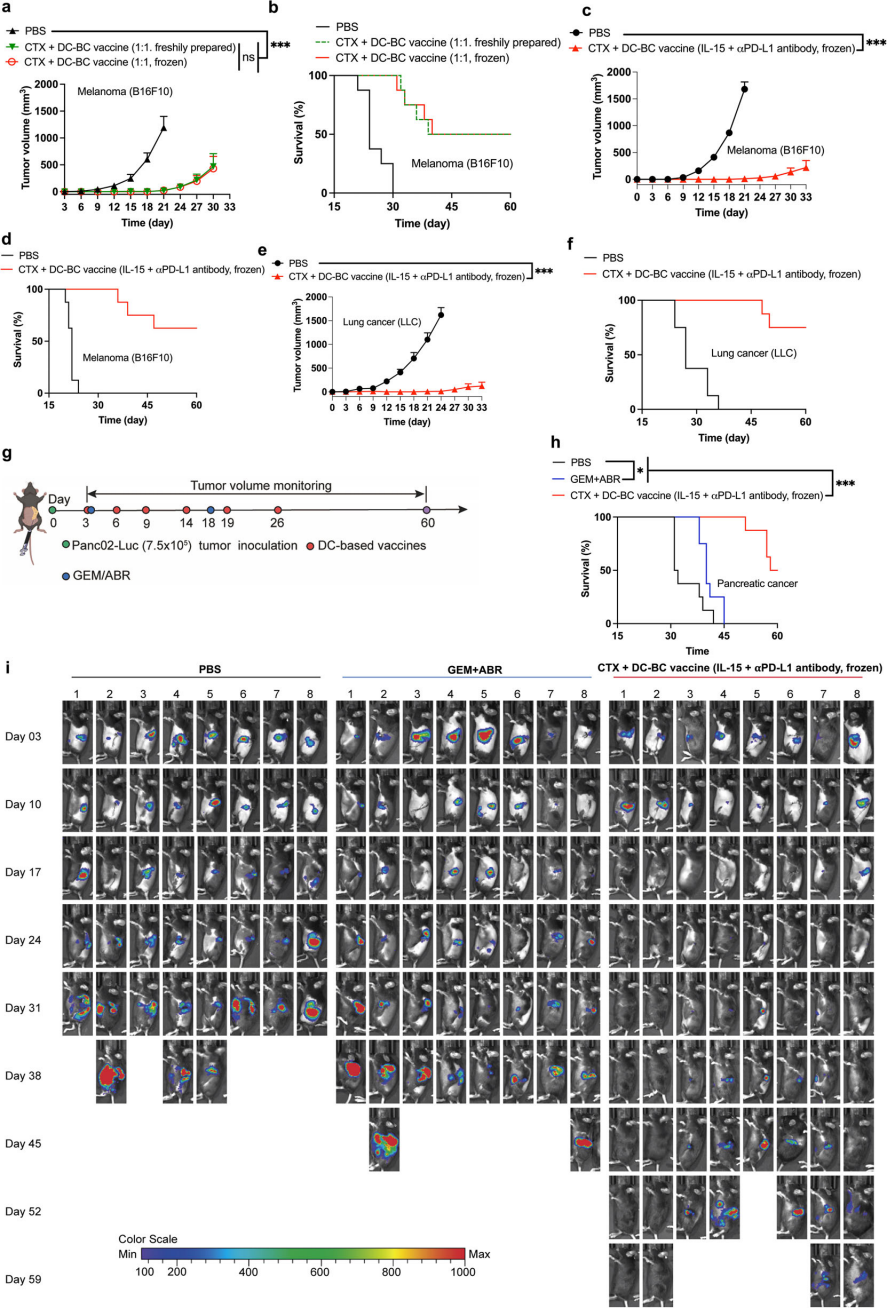
The therapeutic efficacy of cryopreserved DC‐BC vaccines on melanoma, lung cancer and pancreatic cancer mouse models. The treatment regimen in (a—f) is the same as that in Figure 5e. (a,b) The tumor growth and survival curve of tumor‐bearing B16F10 mice treated with fresh prepared DC‐BC vaccines or cryopreserved DC‐BC vaccines. (c,d) The tumor growth and survival curve of tumor‐bearing B16F10 mice treated with cryopreserved DC‐BC vaccines prepared at optimized conditions. (e,f) The tumor growth and survival curve of tumor‐bearing LLC mice (lung cancer) treated with cryopreserved DC‐BC vaccines prepared at optimized conditions. (g) The administration regimen of DC‐BC vaccines on orthotopic pancreatic cancer mouse model (Pan02‐Luc). (h,i) The survival curve and in vivo imaging of the tumor bearing orthotopic pancreatic cancer mice with DC‐BC vaccines. Data are shown as mean ± SEM (n = 8). Statistical significance (* *p* < 0.05, ** *p* < 0.01, and *** *p* < 0.001) was calculated via one‐way ANOVA with a Tukey post hoc test.

The DC‐BC vaccine, formulated by blending DC with B cells, demonstrated promising therapeutic efficacy in various tumor mouse models. Currently, DC vaccines in clinical‐stage research involve a single withdrawal of patient peripheral blood for induction and cryopreservation, thereby ensuring consistency in quality and precise timing of administration. To this end, we designed a study to prepare a DC‐BC vaccine with a sufficient dosage for six administrations, which was then cryopreserved for future use. As a comparison, another group received freshly prepared DC vaccines just prior to each administration. According to our study, it was found that both methods exhibited comparable efficacy, with no statistically significant difference (Figure 6a,b). The results illustrated that in practice, DC‐BC vaccines can be prepared in one time and be cryopreserved for future usage, which is more convenient.

### 1.11 The Therapeutic Efficacy of DC‐BC Vaccines on Lung Cancer Mouse Models and Pancreatic Cancer Mouse Model

In subsequent studies, the combination of a low dose cyclophosphamide with cryopreserved DC vaccines (incubated with nanoparticles and IL‐15/aPD‐L1) was investigated on B16F10 melanoma mouse model and LLC lung cancer mouse model. This preparation method of DC‐BC vaccines resulted in a cure rate of 62.5% on melanoma mouse model and a cure rate of 75% on lung cancer mouse model, respectively (Figure 6c–f). Most tumor‐bearing mice are cured and become tumor free after the treatment with such DC‐BC vaccines, demonstrated an excellent therapeutic efficacy.

To further verify the efficacy of DC‐BC vaccines in a clinically pertinent and the most malignant cancer model, the DC‐BC vaccines was applied to treat orthotopic pancreatic cancer on an orthotopic mouse model. The investigation demonstrated that gemcitabine (GEM) plus Abraxane (ABR) modestly, which is the first‐line clinical treatment method, improved the survival of tumor‐bearing mice compared to the PBS group. However, DC‐BC vaccines at least doubled the survival time of tumor‐bearing mice compared to the PBS group, with two out of eight mice achieving complete remission. This outcome was markedly superior to the first‐line treatment regimen for pancreatic cancer (Figure 6g–i). Considering that orthotopic pancreatic cancer is the most malignant cancer, the therapeutic efficacy of such DC‐BC vaccines is outstanding.

All the therapeutic studies conducted on different tumor‐bearing mouse conclusively demonstrated that the DC‐BC vaccines possess universal applicability and exhibit significant therapeutic efficacy against malignant tumors. These studies provided and new type of DC‐BC cellular vaccines that have much better therapeutic efficacy and paved the way for further potentially clinical translation.

## 2 Discussions

DC vaccine is an important type of therapeutic cancer vaccines. Although one DC vaccine has been approved by FDA for treating prostate cancer, the therapeutic efficacy of such type of DC vaccine is not good enough. The inadequate therapeutic efficacy of DC vaccine is due to 2 reasons: (1) tumor antigens in current DC vaccines are not highly diverse, and thus they cannot overcome the limitations of the high heterogeneity of tumor cells and tumor antigens; (2) The DC vaccines are not efficiently activated in vitro during the preparation of such DC vaccines.

To overcome the problems existing in current DC vaccines, 3 aspects can be further optimized: (1) tumor antigens and their formulation utilized to stimulate DC vaccines; (2) cells sources of DC vaccines; (3) the co‐stimulatory molecules and process of DC vaccines during co‐incubating. By optimizing these factors above, we developed a new DC‐BC vaccine: co‐incubating DC with B cells at the activation process and utilizing NP loaded with whole tumor antigens to prime DC‐BC vaccines. By doing these, an optimal DC‐ BC cancer vaccine was explored and most tumor‐bearing mice can be cured by such DC‐BC vaccines. Thus, the therapeutic efficacy of DC‐ BC cancer vaccines was maximized.

This study showed that the therapeutic efficacy of DC vaccines was significantly improved by using two types of APC (DC and B cells) that can mutually stimulate each other. The enhanced therapeutic efficacy of DC vaccines is due to the crucial roles of different antigen‐presenting cells (APC) in immune responses and their mutual interactions. In addition, NP loaded with whole tumor antigen showed much better effect than multiple neo‐peptides and free tumor lysates.

Herein, we meticulously screened for the optimal dosing concentrations, the optimal co‐incubation times, the optimal cell mixing ratio, and the optimal preparing method of DC‐BC vaccines to achieve their peak efficacy. The vaccines prepared under these conditions effectively inhibited tumor growth. When mixed cells were co‐incubated with nanoparticles + IL‐15, the therapeutic efficacy was further potentiated.

In addition, adding αPD‐L1 antibody into the co‐incubation system can further improve the therapeutic efficacy. Various studies have observed the increased PD‐L1 expression during the in vitro induction process of DC vaccines. Therefore, this study compared three different combination strategies: co‐incubating DC‐BC vaccines together with αPD‐L1 antibody, blocking αPD‐L1 prior to vaccine administration, and co‐injecting DC‐BC vaccines with αPD‐L1 antibody. It was discovered that preparing DC‐BC vaccines by co‐incubating αPD‐L1 antibody, nanoparticles, DC and B cells yielded the best therapeutic results. Additionally, the combination of low‐dose cyclophosphamide with DC‐BC vaccines also demonstrated a notable synergistic effect.

Moreover, the study revealed that DC‐BC vaccines stored at frozen formation exhibited similar therapeutic efficacy with DC‐BC vaccines used immediately after preparation. DC‐BC vaccines also demonstrated favorable biosafety. In multiple tumor‐bearing mouse model, including melanoma, lung cancer and orthotopic pancreatic cancer, the optimal DC‐BC vaccines showed outstanding therapeutic efficacy and demonstrated exceptional cure rates.

More importantly, the mechanisms underlying the interactions between DC and B cells during co‐priming with NP loading whole tumor antigens was explored through conducting a proteomic analysis. The data demonstrated that Toll‐like receptor signaling pathway, NOD‐like receptor signaling pathway, and RIG‐I‐like receptor signaling pathway were involved in DC and B cells co‐activating. In addition, further exploration revealed that MAVS in the RIG‐I and NOD‐like signaling pathways, Ticam1 (TRIF) in the Toll‐like and NOD‐like signaling pathways, Traf3 in the Toll‐like, RIG‐I, and NOD‐like signaling pathways, and Ifnar2 played key roles in promoting the maturation of DC and B cells during co‐priming. Besides, CD40 was increased during the co‐activation. TRIF inhibitor was selected to confirm the function of these above proteins in this co‐activation process. The study illustrated that adding TRIF inhibitor into the co‐incubation system can decrease the therapeutic efficacy of DC‐BC vaccines, demonstrating the significant enhancement in therapeutic efficacy was achieved by the involvement of TRIF.

In summary, a new approach of using two types of APC to prepare DC‐ BC vaccine was presented by this study. It showed much better therapeutic efficacy than using only DC or applying multiple neo‐antigens as tumor antigens for priming. This novel strategy provides a robust experimental and theoretical foundation for further clinical translation and holds promise for advancing DC‐ BC cancer vaccine therapy in the treatment of cancer.

## 3 Materials and Methods

### 3.1 Materials

Lyso‐tracker green, and Hoechst 33342 were purchased from Beyotime Institute of Biotechnology. Cyclophosphamide (CAS 50‐18‐0, Adamas), Resatorvid (TAK‐242, CAS 243984‐11‐4, aladdin), D‐Luciferin potassium salt (CAS 115144‐35‐9, Adamas), and Rhodamine B (CAS 81‐88‐9, Adamas) were purchased from Shanghai Titan Scientific Co.,Ltd. Cell Counting Kit‐8 (Cat. No.: HY‐K0301) were purchased from MedChemExpress. PBS, DMEM medium, 1640 medium, fetal bovine serum, trypsin EDTA solution, and penicillin‐streptomycin solution were purchased from Wuhan Pricella Biotechnology Co.,Ltd. Anti‐PD‐L1 antibody (STARTER, catalog number S0B0593, Clone: 10F.9G2), Pan B Cell Isolation Kit II, mouse (Miltenyi, catalog number 130‐104‐443), LD Columns (Miltenyi, catalog number 130‐042‐901‐1), and auto MACS Running Buffer (Miltenyi, catalog number 130‐091‐221‐1) were obtained from Shanghai Universal Biotech Co., Ltd. IL‐4 (PeproTech, catalog number 214‐14, Source: E. coli), GM‐CSF (PeproTech, catalog number 315‐03, Source: E. coli), M‐CSF (PeproTech, catalog number 315‐02, Source: E. coli), IL‐15 (PeproTech, catalog number 210‐15, Source: E. coli), TNF‐α (PeproTech, catalog number 315‐01A, Source: E. coli), IL‐6 (PeproTech, catalog number 216‐16, Source: E. coli), IL‐1β (PeproTech, catalog number 2111‐11B, Source: E. coli), and IL‐2 (PeproTech, catalog number 212‐12, Source: E. coli) were obtained from Dakewe Biotech Co., Ltd. Zombie Aqua Fixable Viability Kit (BioLegend, catalog number 423102, dilution: 1:200), anti‐mouse CD16/32 (BioLegend, catalog number 101320, clone: 93, dilution: 1:200), APC/Cyanine7 anti‐mouse B220 (BioLegend, catalog number 103224, clone: RA3‐6B2, dilution: 1:200), PerCP/Cyanine5.5 anti‐mouse CD11c (BioLegend, catalog number 117328, clone: N418, dilution: 1:200), PE/Cyanine7 anti‐mouse CD86 (BioLegend, catalog number 159208, clone: A17199A, dilution: 1:200), FITC anti‐mouse I‐A/l‐E (BioLegend, catalog number 107606, clone: M5/114.15.2, dilution: 1:200), APC anti‐mouse CD80 (BioLegend, catalog number 104714, clone: 16‐10A1, dilution: 1:200), PE anti‐mouse‐H‐2 kb (BioLegend, catalog number 116507, clone: AF6‐88.5, dilution: 1:200), APC anti‐mouse CD40 (BioLegend, catalog number 124612, clone: 3/23, dilution: 1:200), PE anti‐mouse CD154 (CD40L) (BioLegend, catalog number 158104, clone: QA17A53, dilution: 1:200), PE anti‐mouse CD274 (B7‐H1, PD‐L1) Antibody (BioLegend, catalog number 124308, clone: 10F.9G2, dilution: 1:200) were purchased from Dakewei Biotech Co., Ltd.

### 3.2 Ethics of Animal Studies

The animal (Approved ethics number 202312A0002) procedures were approved and monitored by the Animal Care and Use Committee of Soochow University. The mice were housed in a specific pathogen‐free (SPF) animal room at the School of Pharmacy, Soochow University, with a constant temperature of 22 ± 1°C, relative humidity of 50 ± 10%, 12‐h artificial light cycle, and automatic ventilation, adhering to the guidelines outlined in the Guide for the Care and Use of Laboratory Animals.

### 3.3 Cell Culture

The cancer cell lines used for the in vivo mouse tumor models included B16F10 cells, Panc02‐Luc cells, and LLC cells. Murine B16F10 cell line (RRID:CVCL_0159), DC2.4 cell line (RRID:CVCL_J409), and LLC cell line (RRID:CVCL_4358) were purchased from the Shanghai Cell Bank, Chinese Academy of Sciences. Murine Panc02‐Luc cell line (RRID:CVCL_A8QL) was purchased from Shanghai Fuheng Biotechnology Co. LTD (Cat No. FH1055). All the cell lines were contamination free. All cells were cultured in DMEM medium with 10% fetal bovine serum and antibiotics. Cells were cultured in an incubator (Thermo Fisher Scientific) at 37°C under an atmosphere of 5% CO_2_ and 90% relative humidity.

### 3.4 Induction of Bone Marrow‐Derived Dendritic Cells

First of all, a C57bl/6J mouse was euthanized and subsequently immersed in 75% ethanol for 10 min. The femurs and tibias were then removed, immersed in 75% ethanol for an additional 2 min, and finally placed in PBS. In a laminar flow hood, using sterile scissors, both ends of the leg bones were cut open and flushed with PBS into a culture dish until the bones turned white. Marrow was collected from four femur and tibia bones, dispersed by pipetting, and filtered through a 70 µm cell strainer into a 50 mL EP tube. The tube was then centrifuged at 1500 rpm for 6 min. Following centrifugation, the supernatant in the centrifuge tube was discarded, and 4 mL of red blood cell lysis buffer was added. The cells were lysed for 3 min, then transferred to a 15 mL centrifuge tube. To terminate the lysis, 6 mL of PBS was added, and the tube was centrifuged again at 1500 rpm for 3 min. After centrifugation, the supernatant was discarded once more, and the cells were washed twice with 4 mL of PBS. To gently resuspend the cells into a cell suspension, 3 mL of complete 1640 medium was added. The cells were divided equally into three 75 mm cell culture dishes, with 9 mL of complete 1640 medium added to each dish. Additionally, 4 µL of GM‐CSF (concentration of 50 µg/mL) and 4 µL of IL‐4 (concentration of 50 µg/mL) were added to achieve a final concentration of 20 ng/mL for both GM‐CSF and IL‐4 in the culture medium. After 3 days of culture, the culture medium was aspirated into a 50 mL EP tube and centrifuged at 800 rpm for 6 min. Differential centrifugation was performed to remove non‐adherent cells. Half of the supernatant was slowly aspirated and discarded, and the cells were redistributed evenly into the culture dishes. To perform a half‐medium change, 5 mL of complete 1640 medium and 2 µL of GM‐CSF (concentration of 50 µg/mL) and 2 µL of IL‐4 (concentration of 50 µg/mL) were added. Culturing was continued. During the seventh to 10th days, the adherent, aggregated cells were gently pipetted to detach. The cell culture medium was collected, centrifuged at 1500 rpm for 6 min, and the supernatant was discarded. The cells were then combined and resuspended in 10 mL of complete 1640 medium.

### 3.5 B Cell Isolation

A C57bl/6J mouse was euthanized and the single cell suspension of splenocytes was collected. The cells were then counted and MACS buffer (40 µL per 10^7^ cells) was added into the sample. The mixture was incubated at 2°C–8°C for 5 min with the Pan B Cell Isolation Kit (Cat. No. 130‐095‐813) to allow for binding of the antibodies to the target cells. Chilled MACS buffer (30 µL per 10^7^ cells) was added to the cell suspension and mixed well. The mixture was then incubated at 2°C–8°C for an additional 10 min to ensure complete binding of the antibodies. An LD column (Cat. No. 130‐042‐901) was pre‐rinsed with 2 mL of MACS buffer to saturate the column and remove any impurities. After rinsing, the sample from step 4 was loaded onto the column. The column was then washed with 2 × 1 mL of MACS buffer to collect the unlabeled, non‐target cells. When the flow through the column stopped, the column was removed from the magnetic separator, and the retained labeled cells (target B cells) were eluted and collected. The collected cells were centrifuged at 1500 rpm for 3 min to concentrate them for further use.

### 3.6 Preparation of Blank Nanoparticles Loaded With Adjuvants Alone

The double emulsion method was used to prepare nanoparticles. The procedure was as follows: First, 300 µL of the adjuvants dissolved in endotoxin‐free water (6 mg/mL) was added to 1 mL of dichloromethane solution (containing 100 mg/mL PLGA) and sonicated for 1 min. Then, 2.5 mL of PVA solution (20 mg/mL was added, followed by 45 s of sonication. The mixture was then dripped into a 50 mL solution containing 5 mg/mL of PVA and stirred at room temperature for 4 h to facilitate nanoparticle solidification. Finally, the supernatant was removed by centrifugation at 13 680 × g and the precipitate was resuspended in 10 mL of 4% trehalose solution. After freeze‐drying for 48 h, the nanoparticles were stored at −20°C for future use. Throughout the preparation process, strict endotoxin‐free procedures were performed to ensure the quality of the nanoparticle vaccine. These nanoparticles were characterized according to the methods described in our previous publications.

### 3.7 Preparation of Nanoparticles Loaded With Water‐Soluble Components in Tumor Tissue Lysates

The methods used to prepare water‐soluble components were as previously reported. Briefly, tumor tissues were put into ultrapure water and repeated freezing‐thaw for at least five times with sonication. And then, the tumor tissue lysates were centrifuged at 5000 for 20 min to get the supernatant. The supernatant was collected as water‐soluble components of tumor lysates.

The double emulsion method was used to prepare nanoparticles. The procedure was as follows: First, 300 µL of the water‐soluble fraction dissolved in endotoxin‐free water (80 mg/mL, with 6 mg/mL poly (I:C) as adjuvant) was added to 1 mL of dichloromethane solution (containing 100 mg/mL PLGA) and sonicated for 1 min. Then, 2.5 mL of PVA solution (20 mg/mL was added, followed by 45 s of sonication. The mixture was then dripped into a 50 mL solution containing 5 mg/mL of PVA and stirred at room temperature for 4 h to facilitate nanoparticle solidification. Finally, the supernatant was removed by centrifugation at 13 680 × g and the precipitate was resuspended in 10 mL of 4% trehalose solution. After freeze‐drying for 48 h, the nanoparticles were stored at −20°C for future use. Throughout the preparation process, strict endotoxin‐free procedures were performed to ensure the quality of the nanoparticle vaccine. These nanoparticles were characterized according to the methods described in our previous publications.

### 3.8 Preparation of Nanoparticles Loaded With Whole Tumor Tissue Lysates

Tumor tissues were lysed and then solubilized with 8 M urea or 6 M guanidine hydrochloride. The solubilized whole tumor tissue lysates were then loaded into nanoparticles. The method used to prepare the nanovaccines was as follows: First, 300 µL of solubilized tumor tissue lysates (80 mg/mL, 6 mg/mL of total adjuvants) was added to 1 mL of dichloromethane solution (containing 100 mg/mL PLGA) and sonicated for 1 min. In NP4 or NP5, 2 mg/mL poly (I:C) + 2 mg/mL CpG7909 + 2 mg/mL CpG 2395 were applied as adjuvants. Then, 2.5 mL of PVA solution at a concentration of 20 mg/mL was added, followed by 45 s of sonication. The mixture was then dripped into a 50 mL solution containing 5 mg/mL of PVA and stirred at room temperature for 4 h to facilitate nanoparticle solidification. Finally, the supernatant was removed by centrifugation at 13 680 × g and the precipitate was resuspended in 10 mL of 4% trehalose solution. After freeze‐drying for 48 h, the nanoparticles were stored at −20°C for future use. Throughout the preparation process, strict endotoxin‐free procedures were performed to ensure the quality of the nanoparticle vaccine. These nanoparticles were characterized according to the methods described in our previous publications.

### 3.9 Preparation of Nanoparticles Loaded With Multiple Neo‐Antigen Peptides

The double‐emulsion method was used to prepare nanoparticles loaded with multiple neoantigen peptides (B16F10‐M36, B16F10‐M05, B16F10‐M27, B16F10‐M20, B16F10‐M24, gp100:44‐59, and TRP2:180‐188). The peptides were solubilized in 8 M urea at a concentration of 2 mg/mL. The total peptide concentration was 14 mg/mL. 300 µL of peptide solution was added to 1 mL of dichloromethane solution (containing 100 mg/mL PLGA) and sonicated for 1 min. Then, 2.5 mL of PVA solution at a concentration of 20 mg/mL was added, followed by 45 s of sonication. The mixture was then dripped into a 50 mL solution containing 5 mg/mL of PVA and stirred at room temperature for 4 h to facilitate nanoparticle solidification. Finally, the supernatant was removed by centrifugation at 13 680 × g and the precipitate was resuspended in 10 mL of 4% trehalose solution. After freeze‐drying for 48 h, the nanoparticles were stored at −20°C for future use. Throughout the preparation process, strict endotoxin‐free procedures were performed to ensure the quality of the nanoparticle vaccine.

### 3.10 Characterization of Nanoparticles

The size and zeta potential of the nanoparticles were determined using dynamic light scattering (DLS) with a Zetasizer Nano‐ZS instrument. For size measurement, the nanoparticles were dispersed in PBS. For zeta potential analysis, the nanoparticles were dissolved in deionized water, under the same conditions. The morphology of the nanoparticles was examined using a transmission electron microscope (TEM) of the HT7700 model. A 20 µL sample of a 0.1 mg/mL PLGA solution was placed onto a copper grid coated with a carbon film, allowed to dry completely, and then observed under the microscope.

### 3.11 Nanoparticle Internalization Studies

An experiment was conducted where 1 × 10^6^ cells, comprising a 1:1 ratio of bone marrow‐derived dendritic cells (BMDCs) and B cells, were seeded into 12‐well plates. Additional wells were designated exclusively for BMDCs and B cells. Each well received Rhodamine B‐encapsulated NP at a concentration of 0.5 mg/ml. Following administration, the cells were incubated for various durations: 0, 6, 12, 24, 36, and 48 h. At each specified time point, cells from three wells were collected by centrifuging at 1500 rpm for 5 min. Subsequently, the cells underwent staining procedures to differentiate between live and dead cells using the Zombie Aqua Fixable Viability Kit. The samples were then incubated with Fc Block to prevent non‐specific antibody binding. Afterward, the cells were stained with anti‐mouse antibodies specific for CD11c (to identify BMDCs) and B220 (to identify B cells). Finally, the stained cells were analyzed using flow cytometry to assess the distribution and characteristics of the cells over time.

### 3.12 Nanoparticle Subcellular Localization Studies

DC2.4 cells, plated at a density of 2.0 × 10^5^ cells per well, were exposed to Rhodamine‐labeled Nanoparticles (NP) for 24 h at 37°C. 30 min prior to the end of the incubation period, the cells were stained with Hoechst 33342 (at a concentration of 5.0 µg/mL, with 1.0 mL added per well) and Lysotracker Green DND‐26 (also at a concentration of 5.0 µg/mL, with 1.0 mL per well) for 20 min. After staining, the cells were thoroughly washed three times with PBS to eliminate any unbound dye or particles. Finally, the cells were observed and analyzed using a Confocal Laser Scanning Microscope (CLSM; Zeiss LSM710).

### 3.13 Analysis of Cellular Cytotoxicity of Nanoparticles, Tumor Lysate, and Cyclophosphamide on BMDC and B Cells

Initially, BMDCs, and B cells were counted. Subsequently, 50 µL of cell suspension containing approximately 3 × 10^4^ cells were pipetted and plated into a 96‐well plate. Following this, a specific quantity of nanoparticles or tumor lysate was weighed and formulated in DMEM basic medium to attain a starting sample concentration of 40 µg/mL. This solution was then serially diluted using a gradient dilution technique to achieve concentrations of 20, 10, 5, 2.5, 1.25, 0.625, 0.32, 0.16, and 0 µg/mL. Fifty microliters of each diluted concentration, repeated in triplicate for accuracy, were added to the previously plated cells in the 96‐well plate. The plate was then incubated in a culture incubator for 24 h. After the incubation period, 10 microliters of CCK8 reagent were dispensed into each well, and the incubation was continued for an additional 2 h. Thereafter, the absorbance values at a wavelength of 450 nm were recorded for each well. In parallel, cyclophosphamide was prepared at concentrations ranging from 16 to 0 mM (16, 8, 4, 2, 1, 0.5, 0.25, 0.125, 0.065, and 0 mM) and added to corresponding cells, following the aforementioned procedures to assess cytotoxicity.

### 3.14 Measurement of Immune Indicators at Various Time Points and Concentrations of Nanoparticles Inducing BMDCs or a Mixture of BMDCs and B Cells (1:1 ratio)

Pre‐induced BMDCs or pre‐isolated B cells were accurately counted and plated in a 12‐well plate at a density of 1 × 10^6^ cells per well. Specifically, for BMDC or B cell‐related experiments, only 1 × 10^6^ BMDCs or B cells were plated per well, whereas for experiments involving a mixture of BMDCs and B cells (1:1 ratio), 5 × 10^5^ cells of each type were plated. Immediately following cell plating, the nanoparticles were introduced (0.5 mg/mL), and the cells were incubated for durations of 1, 4, 6, 8, 12, 24, 48, and 72 h. At each specified time point, cells from three replicate wells were collected and centrifuged at 1500 rpm for 5 min, preparing them for subsequent staining procedures. Based on the outcomes of these initial experiments, a time point demonstrating optimal immune indicator performance was chosen for further investigation into the effects of varying nanoparticle concentrations. The plating procedure remained unchanged, with nanoparticles added to each well at concentrations of 0, 0.1, 0.3, 0.5, 0.8, 1.0, and 1.5 mg/mL. Each concentration was tested in triplicate. At the designated time point, the cells were collected and centrifuged at 1500 rpm for 5 min, ready for the staining steps to follow. For flow cytometry antibody staining of pure BMDC cells, the Zombie Aqua Fixable Viability Kit was utilized to discriminate between live and dead cells. Subsequently, the samples were incubated with Fc Block to prevent non‐specific binding, and then stained with anti‐mouse antibodies targeting CD11c, CD86, CD80, I‐A/I‐E, and H‐2 k^b^. Finally, the stained cells were subjected to flow cytometry analysis. For the antibody panel for flow cytometry staining of mixed BMDC and B cells, the Zombie Aqua Fixable Viability Kit was again utilized to discriminate between live and dead cells. The samples were then incubated with Fc Block, stained with anti‐mouse antibodies targeting B220, CD11c, CD86, CD80, I‐A/I‐E, and H‐2 k^b^, and subjected to flow cytometry analysis. In terms of flow cytometry‐based antibody staining to evaluate the expression of CD40 and CD40L in a combined population of BMDC and B cells, the Zombie Aqua Fixable Viability Kit was used to discriminate live and dead cells. The samples were then incubated with Fc Block, stained with anti‐mouse antibodies targeting B220, CD11c, CD86, I‐A/I‐E, CD40, and CD154 (CD40L), and analyzed by flow cytometry.

### 3.15 The Investigation of DC Activations and T Cells Activations

In the studies, macrophages are BMDM and DC are BMDC. After co‐incubating BMDC + B cells (1:1 ratio, DC‐BC) or BMDC + BMDM cells (1:1 ratio, DC‐DM) with NP5 (0.5 mg/mL) for 36 h, the expression of immune markers in different cells were measured. At the designated time point, the cells were collected and centrifuged at 1500 rpm for 5 min and them were stained with flow cytometry antibodies. For flow cytometry antibody staining, the Zombie Aqua Fixable Viability Kit was utilized to discriminate between live and dead cells. Subsequently, the samples were incubated with Fc Block to prevent non‐specific binding, and then stained with anti‐mouse antibodies targeting CD11c, CD86, CD80, I‐A/I‐E, and H‐2 k^b^. Finally, the stained cells were subjected to flow cytometry analysis.

In terms of T cell proliferation studies, BMDC + B cells (1:1) or BMDC + BMDM (1:1) were co‐incubated with NP 5 for 36 h. And then, the mixtures were combined with magnetically sorted T cells at a 1:500 ratio and seeded into 96‐well plates. CCK‐8 reagent was added at 12, 24, 48, and 72 h, and the absorbance at 450 nm was measured to calculate T cell proliferation.

In the in vitro investigation of T cell activation studies, BMDC + B cells (1:1 ratio, DC‐BC) or BMDC + BMDM cells (1:1 ratio, DC‐DM) were co‐incubated with NP5 for 36 h. DC‐BC or DC‐DM vaccines were then co‐cultured with sorted T cells at a ratio of 1:500 (APC vaccine cells: T cells). The positive rates of CD69 and CD25 on T cells were measured at 3, 6, 12, and 24 h. At the designated time point, the cells were collected and analyzed with flow cytometry studies. The Zombie Aqua Fixable Viability Kit was utilized to discriminate between live and dead cells. Fc Block was applied to prevent non‐specific binding, and anti‐mouse CD25 and CD69 antibodies were used to label the activated T cells.

In the in vivo investigation of T cell activation studies, B16F10 tumor‐bearing mice were subcutaneously injected with one million DC‐BC or DC‐DM vaccine cells. The T cells in draining lymph nodes were analyzed and the amount of CD69 and CD25 positive T cells were quantified at 24, 48 and 72 h post‐injection. The flow cytometry assay procedure is the same as described in the previous method.

### 3.16 Measurement of PD‐L1 Expression After Co‐Incubating Nanoparticles With BMDCs and B Cells

Pre‐induced BMDCs or pre‐isolated B cells were accurately counted and plated in a 12‐well plate at a density of 1*10^6^ cells per well (for mixed cultures of BMDC and B cells, 5*10^5^ cells of each type were plated). Immediately upon plating, the cells were treated with nanoparticles at a concentration of 0.5 mg/mL. After a 48‐h incubation period, cells from three replicate wells were harvested and centrifuged at 1500 rpm for 5 min. Subsequently, the cells underwent staining procedures. To differentiate live from dead cells, the Zombie Aqua Fixable Viability Kit was employed. Following this, the samples were incubated with Fc Block to hinder non‐specific antibody binding and then stained with anti‐mouse antibodies specific for B220, CD11c, and CD274. Ultimately, the stained cells were analyzed using flow cytometry.

### 3.17 Preparation of DC‐BC Vaccines

Pre‐induced BMDCs and pre‐isolated B cells were gathered and counted. In a 75 mm cell culture dish, for DC vaccines consisting solely of BMDCs, 5 × 10^6^ BMDCs were added per dish. For DC vaccines containing both BMDCs and B cells, a mixture of 2.5 × 10^6^ BMDCs and 2.5 × 10^6^ B cells was added per dish. The medium was then adjusted to a total volume of 10 mL, and the appropriate nanoparticles were introduced at a concentration of 0.5 mg/mL. Depending on the vaccine formulation, specific cytokines were also added at the following concentrations: IL‐15 at 10 ng/mL, GM‐CSF at 20 ng/mL, 2‐BP at 50 mM, Flt3‐L at 100 ng/mL, IL‐1β at 5 ng/mL, IL‐6 at 160 ng/mL, PEG‐2 at 2 mg/mL, TNF‐α at 5 ng/mL, and αPD‐L1 antibody at 1.5 µg/mL. After 48 h of incubation, the suspended cells were harvested by centrifuging at 1500 rpm for 5 min. The adherent cells were subsequently digested using trypsin, combined with the suspended cells, and resuspended in PBS to produce the DC vaccine. If cryopreservation was necessary, the combined adherent and suspended cells were mixed with serum‐free cell freezing medium for storage. Prior to use, the cells were thawed at 37°C, centrifuged at 2000 rpm for 5 min, the supernatant was discarded, and the cells were re‐suspended in PBS to yield the final DC vaccine.

### 3.18 Establishment of Tumor‐Bearing B16F10 and LLC Mouse Models

Female C57BL/6J mice, aged between 6 and 8 weeks, were acquired and utilized for the establishment of subcutaneous tumors. Tumor cells in the logarithmic growth phase and in optimal condition were injected into the dorsal region of the mice, close to the thigh, in a volume of 100 µL. Each mouse received 1.5 × 10^5^ B16F10 cells (Each mouse in the LLC model was injected with 2 × 10^6^ LLC cells). Subsequent to the injection, the mice were allocated into groups according to the pre‐established experimental protocol. The DC vaccines were administered subcutaneously to the mice on days 3, 6, 9, 14, 19, and 26 following tumor inoculation. In specific experiments designed to inhibit tumor growth, myelosuppression was induced 48 h post‐tumor inoculation by administering cyclophosphamide intraperitoneally at a dosage of 100 mg/kg.

### 3.19 Establishment of Panc02‐Luc Orthotopic Pancreatic Cancer Mouse Model

Panc02‐Luc cells, which were in optimal condition and at the logarithmic growth phase, were harvested at a concentration of 1.5 × 10^7^ cells per milliliter. These cells were then mixed with Matrigel in a 1:1 ratio by volume and kept on ice for future use. 6 to 8 week‐old C57BL/6J mice underwent hair removal on their left abdominal area and were anesthetized via intraperitoneal injection of 200 µL of chloral hydrate at a concentration of 35 mg/mL. A small incision was made on the left abdominal wall to expose the pancreas, which was then carefully exteriorized for the procedure. A total of 50 µL of the pre‐prepared Panc02‐Luc cell suspension mixed with Matrigel was injected into the tail of the pancreas. The pancreas was allowed to rest for 3 min before being gently repositioned within the abdominal cavity. The surgical site was closed by suturing the muscular layer first, followed by the skin layer. The wound was then coated with erythromycin ointment to prevent infection, and the mouse was placed in a clean cage for recovery. 48 h post‐surgery, the successful establishment of the tumor model was confirmed using the IVIS Spectrum Lumina III (PerkinElmer). Following this, myelosuppression was induced by intraperitoneal injection of cyclophosphamide at a dosage of 100 mg/kg. DC vaccines (The dosage for each mouse is 1 × 10^6^ cells) were administered subcutaneously on days 3, 6, 9, 14, 19, and 26 after tumor inoculation. The chemotherapy group (GEM/ABR) received intravenous injections on days 3 and 11 with gemcitabine (GEM) at a dose of 35.0 mg/kg. Bioluminescence images of the mice were acquired every 7 days after the injection of the DC vaccine using the IVIS Spectrum Lumina III (PerkinElmer), and the number of natural deaths in each mouse group during the experimental period was meticulously documented.

### 3.20 Analysis of Potential Toxicities of Vaccines by Investigating Various Blood Biochemistry Indicators and Hematoxylin‐Eosin Staining

A subcutaneous B16F10 tumor model was established, followed by treatment with mature dendritic cell vaccines (The dosage for each mouse is 1 × 10^6^ cells) that were induced through various methodologies. 3 days after the final vaccine administration, blood was collected via ocular bleeding and placed into EP tubes containing heparin sodium as an anticoagulant. The blood samples were then centrifuged at 5000 rpm for 10 min to separate the serum, which was subsequently stored at −80°C for subsequent biochemical analysis. Simultaneously, the heart, liver, spleen, lungs, and kidneys were harvested from the animals and fixed in 4% paraformaldehyde solution. These tissues were then embedded in paraffin for sectioning, stained with hematoxylin and eosin (HE), and examined under a fluorescence upright microscope to assess any pathological morphological changes in the stained tissue sections.

### 3.21 Analysis of DC and B Cells Interactions During Co‐Incubating by Proteomics

Collect the induced Bone Marrow‐Derived Dendritic Cells (BMDCs) and isolated B cells, and perform cell counting. Place 10 million cells (5 million each of BMDCs and B cells) per 100 mm diameter dish. The groups are divided as follows: BMDCs + NP5, B cells + NP5, BMDCs + B cells + NP5, and BMDCs + B cells + NP5 (with flow cytometry sorting for BMDCs and B cells). Add 10 mL of complete medium containing 0.5 mg/mL NP5 to each dish and incubate in a cell culture incubator for 36 h. After 36 h, harvest the cells and centrifuge at 1500 rpm for 5 min. Wash the cells twice with PBS and store in −80°C. For the BMDCs + B cells + NP5 group (with flow cytometry sorting for BMDCs and B cells), after centrifugation, add the Zombie Aqua Fixable Viability Kit. Subsequently, incubate the samples with Fc Block to prevent non‐specific antibody binding, and then stain with anti‐mouse antibodies specific for B220 and CD11c. Finally, sort the BMDCs and B cells using flow cytometry, centrifuge to remove the supernatant, and store at −80°C. Send the samples to Shanghai Bioprofile Technology Co., Ltd. for analysis.

### 3.22 Statistical analysis

All statistical analyses were performed using GraphPad Prism software version 8.3.0 and R software version 4.1. Data were analyzed using t‐test, and chi‐square test based on data type and normality distribution. Error bars represent standard deviation (SD) or standard error of the mean (SEM). A *p*‐value < 0.05 was considered statistically significant: ^*^ *p* < 0.05; ^**^ *p* < 0.01; ^***^ *p* < 0.001. Unless otherwise stated, asterisks indicate statistical comparisons with the control group.

## Author Contributions

M.L. conceived and designed the study. M.L., L.D., and J.W. conducted nanoparticle preparation, X.X., X.C., J.W., L.D., and Y.L. conducted animal studies and flow cytometry studies. M.L., J.Z., and X.X. analyzed the data and drew the figures. M.L, and J.Z. provided the resources for this study. X.X. wrote the draft and M.L. revised the manuscript. All authors reviewed and approved the final version.

## Funding

The research was funded by Suzhou Ersheng Biopharmaceutical Co., Ltd., Suzhou, Jiangsu, People's Republic of China, and Wuxi Boston Biopharmaceutical Co., Ltd., Wuxi, 214125, People's Republic of China.

## Conflicts of Interest

The authors declare no conflict of interest.

## Supporting information




**Supporting File**: advs74280‐sup‐0001‐SuppMat.pdf.

## Data Availability

The data supporting the findings of this study are included in the paper and its extended data. All other relevant data are available from the corresponding author upon reasonable request.
