# Supplementary material for: Redefining Dendritic Cell Vaccines: Synergistically Co‐priming DC and B Cells With Nanoparticles Loading Whole Cell Antigens Maximizes the Efficacy of DC Vaccines
Source: Adv Sci (Weinh). 2026 Feb 11;13(22):e10615. doi: 10.1002/advs.202510615 (PMC13088298; doi:10.1002/advs.202510615)
Supplement: Supplementary file 1 — Supporting File: advs74280‐sup‐0001‐SuppMat.pdf. [file ADVS-13-e10615-s001.pdf]

Redefining Dendritic Cell Vaccines: Synergistically Co-priming DC and B Cells with Nanoparticles Loading Whole Cell Antigens Maximizes the Efficacy of DC Vaccines

Xiangxiang Xu[1,2,3,4,5#], Xianlan Chen[1,2#], Jin Wang, Yuhan Liu[1,2], Sidra Mustafa[1], Lu Diao[1,2,3,4], Rongrong Zhao[1,2], Haiyang Chi[1,2], Kang Hu[2,6,7], Jiashan Zhu[2,6,7], Jun Zhao[2,6,7], Mi Liu[1,2,3,4,8]*

[1] Department of Pharmaceutics, College of Pharmaceutical Sciences, Soochow University, Suzhou, 215123, People's Republic of China

[2] Institute of Minimally Invasive Thoracic Cancer Therapy and Translational Research, Soochow University, Suzhou, Jiangsu, 215123, People's Republic of China

[3] Ersheng Biopharmaceutical Co., Ltd., Suzhou, Jiangsu, 215000, People's Republic of China

[4] Wuxi Boston Biopharmaceutical Co., Ltd., Wuxi, 214125, People's Republic of China

[5] Affiliated Changshu Hospital of Nantong University, Changshu, 215500, China

[6] Institute of Thoracic Surgery, The First Affiliated Hospital of Soochow University, Soochow University, Suzhou, Jiangsu, 215123, People's Republic of China

[7] Department of Thoracic Surgery, The First Affiliated Hospital of Soochow University, Soochow University, Suzhou, Jiangsu, 215123, People's Republic of China

[8] Jiangsu Province Engineering Research Center of Precision Diagnostics and Therapeutics Development, Soochow University, Suzhou 215123, China, People's Republic of China.

[#] Contribute equally to this study

[*] Corresponding author, Mi Liu, Email: mi.liu@suda.edu.cn

Table S1

NP	Zeta Potential (mV, ±SD)	Size (nm, ±SD)	Loading capacity (μg/mg proteins)
Blank NP	-28.89±0.23	260.95±11.96	0
Neo-antigen NP	-26.94±0.55	287.39±10.63	49.04±5.59
NP loading only water-soluble lysates	-27.11±0.40	291.19±9.98	130.42±6.69
NP1	-26.33±0.51	295.41±12.32	135.11±7.55
NP2	-27.70±0.45	300.78±1038	132.22±6.15
NP3	-26.92±0.39	279.09±11.03	130.87±8.62
NP4	-25.99±0.61	282.33±14.09	133.38±8.10
NP5	-26.58±0.37	293.87±11.09	131.89±6.91

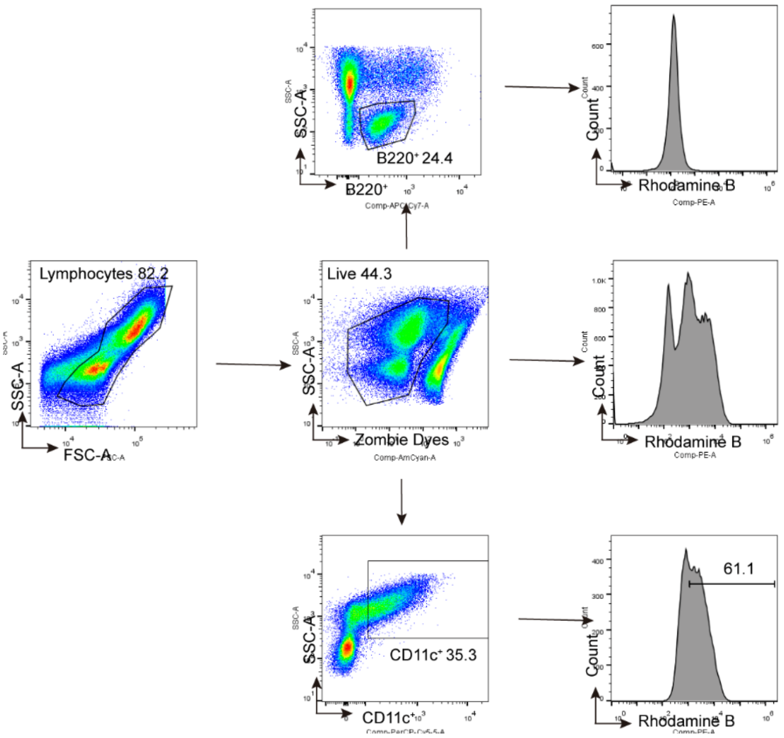

Figure S1. Flow cytometry gating strategy for detecting uptake of rhodamine-labeled nanoparticles by pre-mixed BMDCs and B cells.

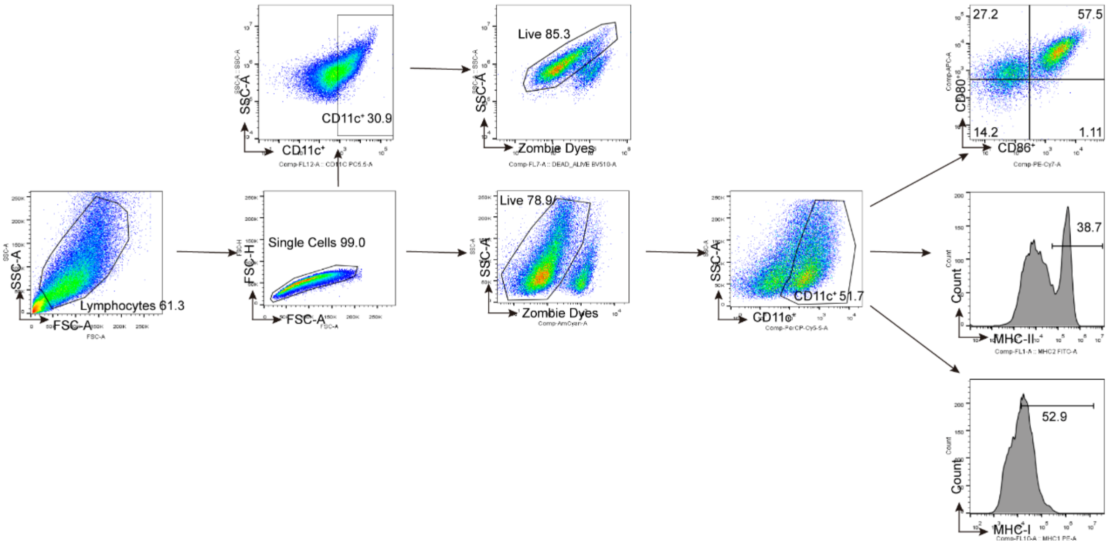

Figure S2. Flow cytometry gating strategy for detecting immune markers after nanoparticles incubation with BMDCs.

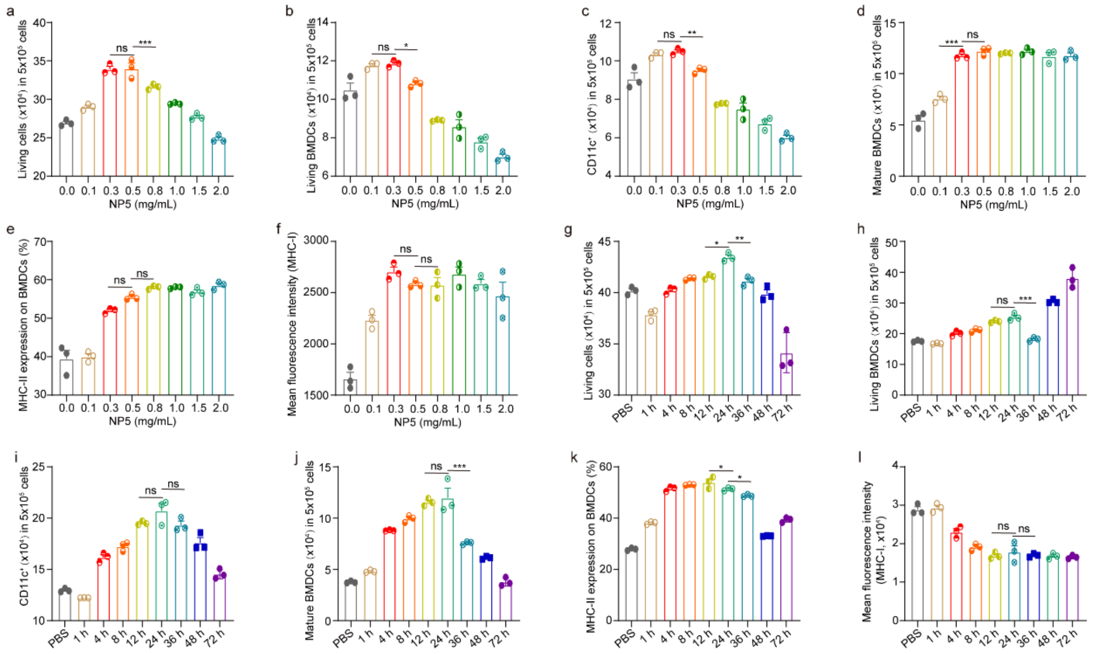

Figure S3. Different concentrations of nanoparticles or the same concentration at different time induced BMDC related immune indexes. After incubation of BMDC with NP5 at no concentration for 24 hours, total cell survival of BMDC incubated in 500 000 cells (a); the number of surviving BMDC in 500 000 cells (b); the number of CD11c-positive cells surviving in 500 000 cells (c); number of mature BMDC in 500 000 cells (d); percentage of MHC-II expression (e); MHC-I relative fluorescence intensity statistics (f). After incubation of BMDC with NP5 at 0.5mg/mL for different times, the total number of cell survival in 500 000 cells (g); the number of surviving BMDC in 500 000 cells (h); the number of CD11c-positive cells surviving in 500 000 cells (i); the number of mature BMDC in 500 000 cells (j); the percentage of MHC-II expression (k); MHC-I relative fluorescence intensity statistics (l). Data are shown as mean ± SEM (n = 3). Statistical significance (* P<0.05, ** P<0.01, and *** P<0.001) was calculated via one-way ANOVA with a Tukey post hoc test.

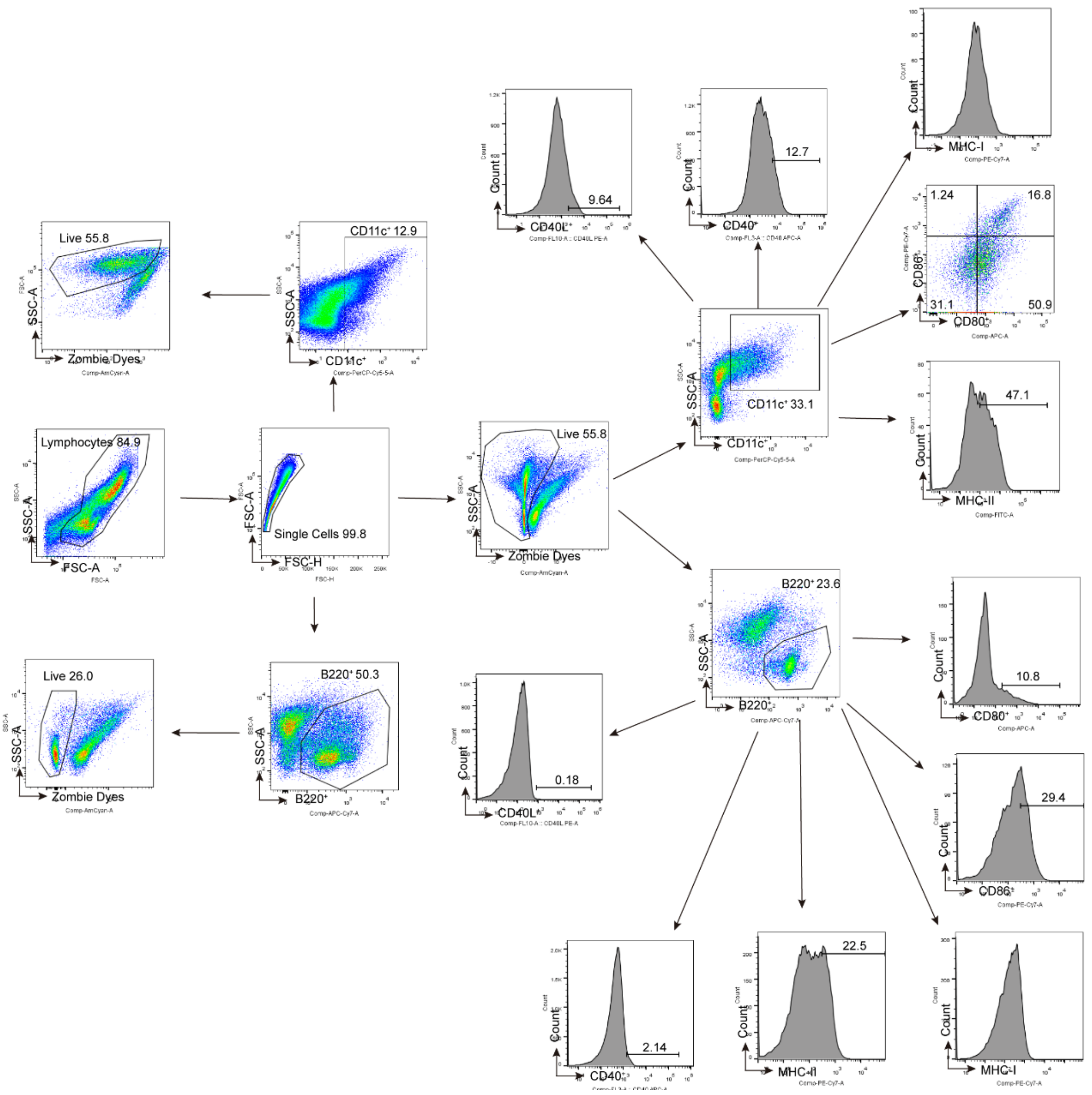

Figure S4. Flow cytometry gating strategy for detecting immune markers after incubating nanoparticles with pre-mixed BMDCs and B Cells.

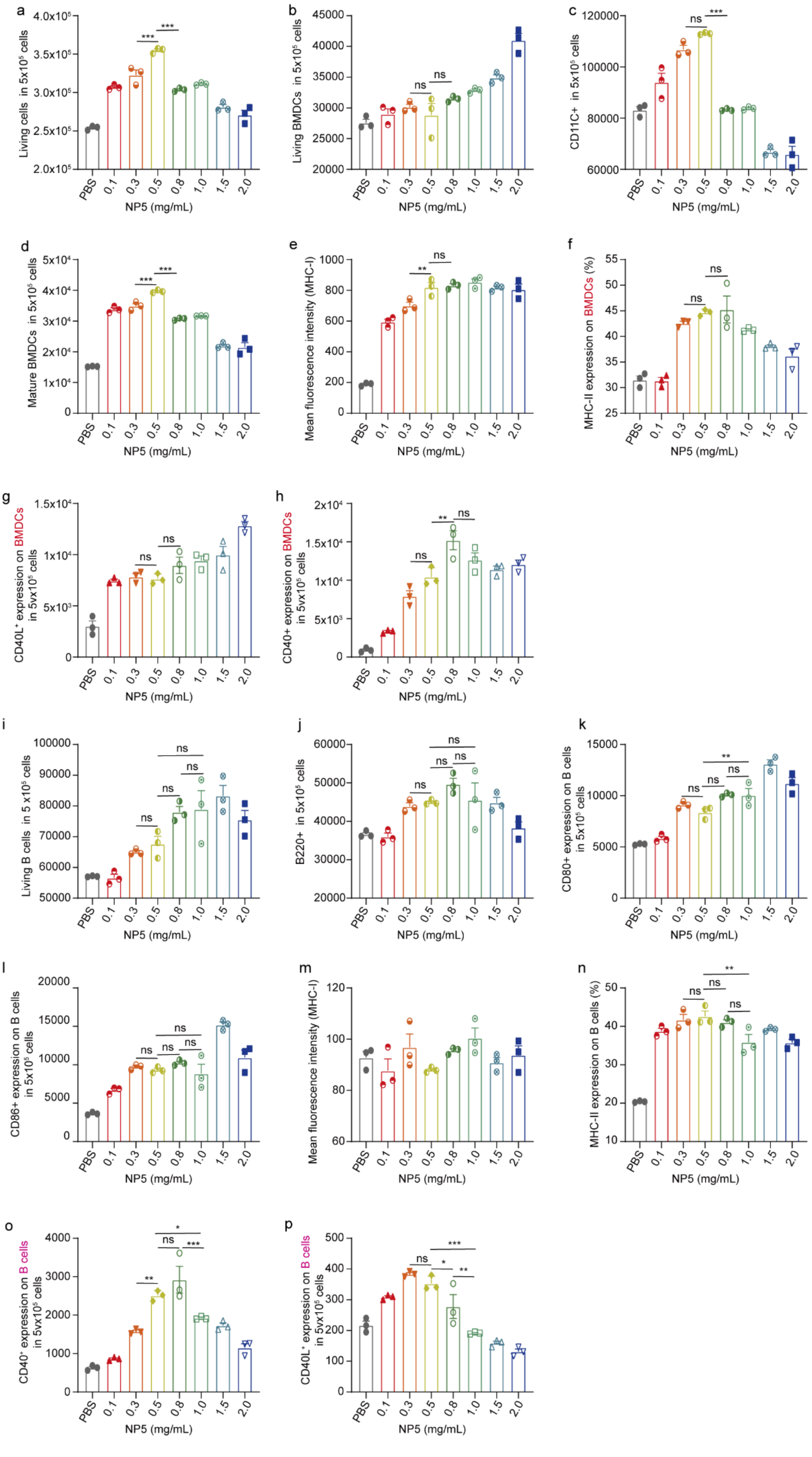

Figure S5. Detection of immunological parameters related to BMDC and B cells (1:1

premixed) induced by nanoparticles of varying concentration at the same time points. Incubation of BMDCs and B cells with NP5 at the varying concentration for 24 h in 500 000 cells: a) total cell survival; b) the number of surviving BMDCs; c) survival number of CD11c positive BMDCs; d) number of CD80 and CD86 double positive BMDCs; e) MHC-I relative fluorescence intensity statistics of BMDCs; f) MHC-II expression percentage of BMDCs; g) survival number of CD40L-positive cells; h) the surviving number of CD40 positive cells. Incubation of BMDCs and B cells with NP5 at varying concentration for 24 hours; i) number of B cell survival in 500 000 cells; j) survival number of B220 positive cells; k) the number of CD80-positive B cell; l) the number of CD86-positive B cell; m) MHC-I relative fluorescence intensity statistics of B cells; n) percentage of MHC-II expression on B cells; o) the surviving number of CD40 positive cells; p) Survival number of CD40L-positive cells. Data are shown as mean ± SEM (n = 3). Statistical significance (* $P<0.05$, ** $P<0.01$, and *** $P<0.001$) was calculated via one-way ANOVA with a Tukey post hoc test.

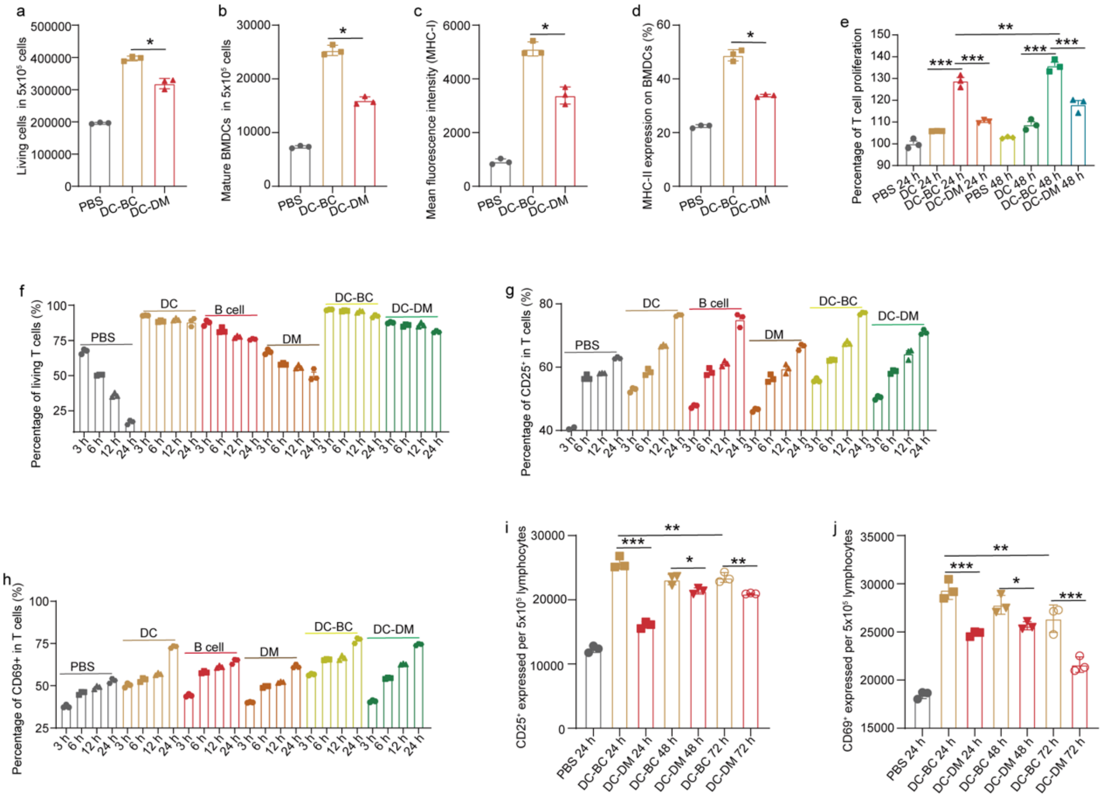

Figure S6. Co-incubating DC + B cells with antigens improve the activation of DC and T cells, comparing with single antigen-presenting cells or DC + macrophages. a) The quantifying analysis of all survival cells after co-incubating DC + B cells (1:1 ratio, DC-BC) or DC + macrophage (1:1 ratio, DC-DM) with NP5 for 36 hours. **b**) The quantifying analysis of CD80$^+$ and CD86$^+$ in BMDCs after co-incubating DC + B cells (1:1 ratio, DC-BC) or DC + macrophages (1:1 ratio, DC-DM) with NP5 for 36 hours. **c**) The analysis of MHC I relative fluorescence intensity statistics of BMDCs after co-incubating DC + B cells (1:1 ratio, DC-BC) or DC + macrophage (1:1 ratio, DC-DM) with NP5 for 36 hours **d**) The MHC II expression percentage in BMDC after co-incubating DC + B cells (1:1 ratio, DC-BC) or DC + macrophages (1:1 ratio, DC-DM) with NP5 for 36 hours. **e**) The proliferation of T cells measured by CCK8 assay at different time points after activating naïve T cells with various vaccines *in vitro*. **f**) The percentage of living T cells in the total T cell population after activating naïve T cells with various vaccines *in vitro*. **g**) The percentage of CD25$^+$ in the T cell population after activating naïve T cells with various vaccines *in vitro*. **h**) The percentage of CD69$^+$ in the T cell population after activating naïve T cells with various vaccines *in vitro*. **i**) The number of CD25$^+$ T cells per 0.5 million lymph node T cells *in vivo* (Mice were subcutaneously injected

with one million vaccine cells and the $CD25^+$ T cells from draining lymph nodes were investigated at 24, 48 and 72 h post-injection). **j**) The Number of $CD69^+$ T cells per 0.5 million lymph node cells *in vivo* (Mice were subcutaneously injected with one million vaccine cells and the $CD25^+$ T cells from draining lymph nodes were investigated at 24, 48 and 72 h post-injection). Data are shown as mean ± SEM (n = 3). Statistical significance (* $P<0.05$) was analyzed via one-way ANOVA.

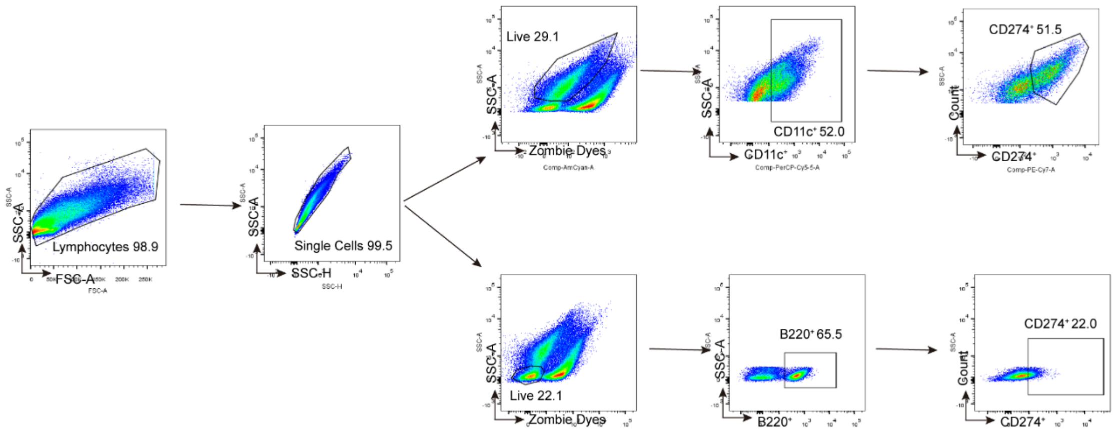

Figure S7. Flow cytometry gating strategy for detecting PD-L1 expression after incubating nanoparticles with pre-mixed BMDCs and B cells.

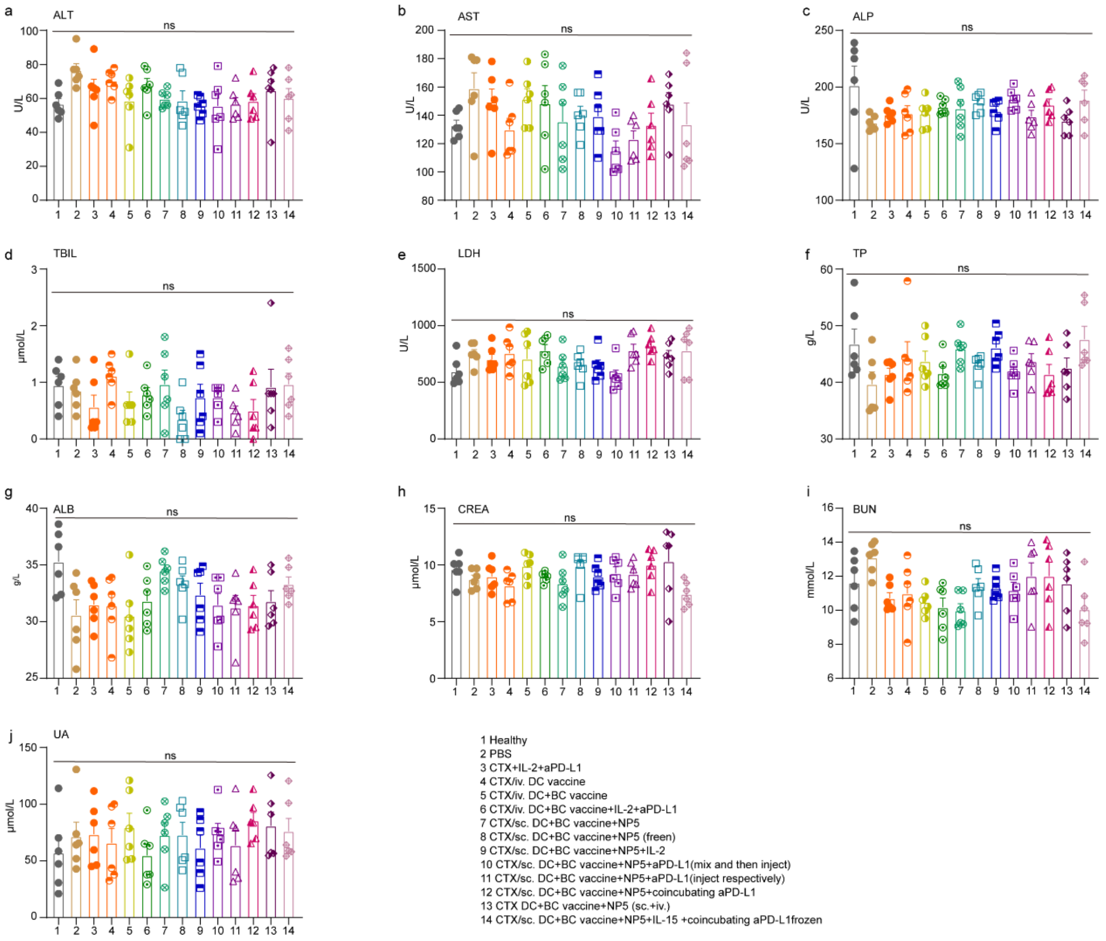

Figure S8. Analysis of potential toxicities of DC+BC vaccine by measuring various biochemical indicators in blood of tumor-bearing mice treated with DC+BC vaccines. a-j, Changes of biochemical indicators in blood of tumor-bearing mice treated with different DC vaccines. Data are presented as Mean ± SD, no significant difference was observed among different groups.

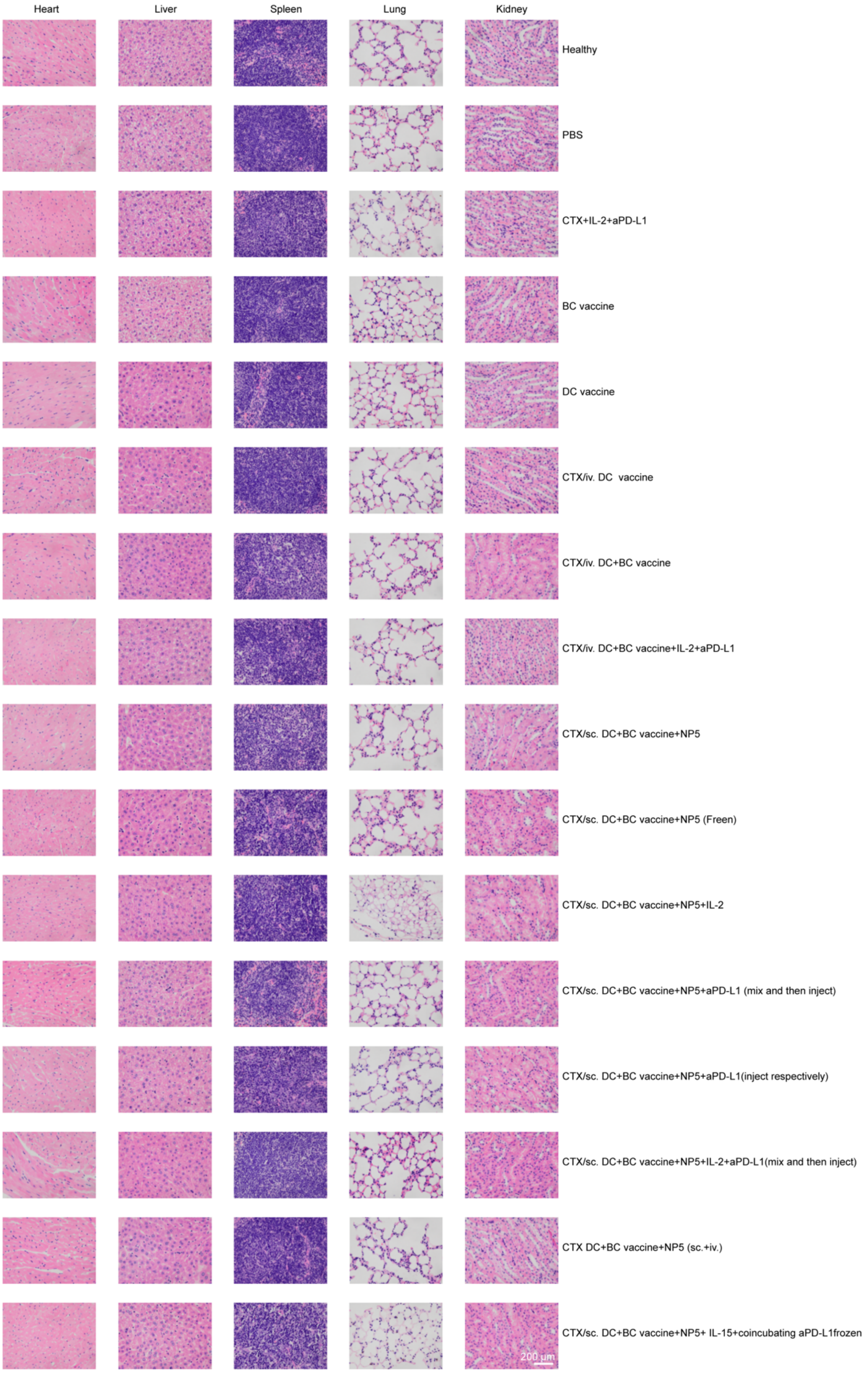

Figure S9. The results of H&E study conducted on sample of heart, liver, spleen, lung and kidney from mice treated with DC+BC vaccine.
